# Mutation-Aware Machine Learning Framework for Predicting Binding Affinity of Nirmatrelvir Analogs Targeting Coronavirus Main Proteases

**DOI:** 10.3390/molecules31172949

**Published:** 2026-08-22

**Authors:** Md Saidur Rahman, Md Mehedi Hasan, Shahidul M. Islam

**Affiliations:** Department of Chemistry, Delaware State University, Dover, DE 19901, USA; mrahman25@students.desu.edu (M.S.R.); mhasan23@students.desu.edu (M.M.H.)

**Keywords:** MERS-CoV, main protease (Mpro), Nirmatrelvir analogues, mutation-aware machine learning, binding-affinity prediction, ensemble learning, drug-resistance mutations, Boltz-2

## Abstract

The emergence of resistance-associated mutations in coronavirus main protease (Mpro) poses a significant challenge to the development of broad-spectrum antiviral therapeutics. In this study, we improved and accelerated a mutation-aware machine learning (ML) framework to predict the binding score of Nirmatrelvir analogue ligands against wild-type and mutant MERS-CoV Mpro. A library of 15,889 Nirmatrelvir derivatives generated through systematic scaffold modification was docked against the wild-type and five variants of the Mpro, producing a total of 95,334 structural and docking score datasets of these protein–ligand complexes. During the ML model development phase, ligand effects were learned from RDKit molecular descriptors and graph-based representations, and the mutation-induced effects were captured through delta-encoded physicochemical properties (hydrophobicity, charge, aromaticity, and polarity) of the active-site residues. Among the evaluated models, the CatBoost regressor tree-based algorithm achieved the lowest mean absolute error (MAE) value of 0.23 Kcal/mol and an R^2^ of 0.87. Further improvement was achieved by creating a weighted ensemble model combining the CatBoost regressor, XGBoost and LightGBM regressor, resulting in a prediction accuracy with a MAE of 0.19 Kcal/mol and an R^2^ of 0.90 relative to docking scores. Model robustness was further evaluated through random-, ligand group- and scaffold group- K-fold cross-validation along with their Y-randomization. Moreover, the models were also tested with a new set of 1000 structurally diverse compounds. SHAP analysis was conducted, which identified 20 molecular descriptors critical for accurate predictions. The ensemble model accurately predicted the binding affinities of Nirmatrelvir and its four analogues (E1–E4), reproducing the experimental pIC50 trend and correctly identifying the most potent inhibitors. The ensemble model also showed consistent performance across all MERS-CoV Mpro variants, S147Y, S142G, L144A, S142G/S147Y, and S142G/L144A/S147Y, demonstrating its potential for rapidly discovering mutation-resistant antiviral drugs.

## 1. Introduction

Nearly two decades after the first identification of SARS-CoV, a related betacoronavirus, SARS-CoV-2, emerged and caused the COVID-19 pandemic [1]. The global case-fatality rate of SARS-CoV-2 was approximately 2–3%, varying by geographic region, time period, and healthcare capacity, but the pandemic resulted in around 780 million confirmed cases and more than 7 million deaths worldwide [2]. Another coronavirus, MERS-CoV, first identified in Saudi Arabia and Jordan in 2012, remains a persistent zoonotic pathogen with a high fatality rate of about 36% [3], making it the most lethal among known human coronaviruses. In 2025, the World Health Organization (WHO) reported 19 laboratory-confirmed MERS-CoV cases globally, including four deaths, with most cases occurring in Saudi Arabia and a few in France [4]. While several potent inhibitors have been developed targeting the SARS-CoV-2 main protease (Mpro), such as Nirmatrelvir [5], Ibuzatrelvir [6], and Ensitrelvir [7], the effectiveness of such inhibitors can be compromised by mutations in the viral protease [8,9]. Both computational and experimental studies indicate that even single amino acid substitutions in coronavirus Mpro can significantly influence inhibitor binding and protein stability, highlighting the need for mutation-aware approaches in antiviral drug design [8,9,10,11,12]. For example, Hu et al. [13] identified 102 naturally occurring mutations in SARS-CoV-2 Mpro, including 12 residues within the Nirmatrelvir-binding region, and found that 22 substitutions at five key residues (S144, M165, E166, H172, and Q192) resulted in a substantial reduction in enzymatic activity (greater than a 10-fold decrease in kcat/Km and an increase in Ki). Similarly, Krismer et al. [14] reported resistance-associated mutations in the catalytic site of MERS-CoV Mpro, including S142G, S142R, and S147Y, further emphasizing the urgent need to develop new therapeutic strategies against both SARS-CoV-2 and MERS-CoV.

**Figure 1:**
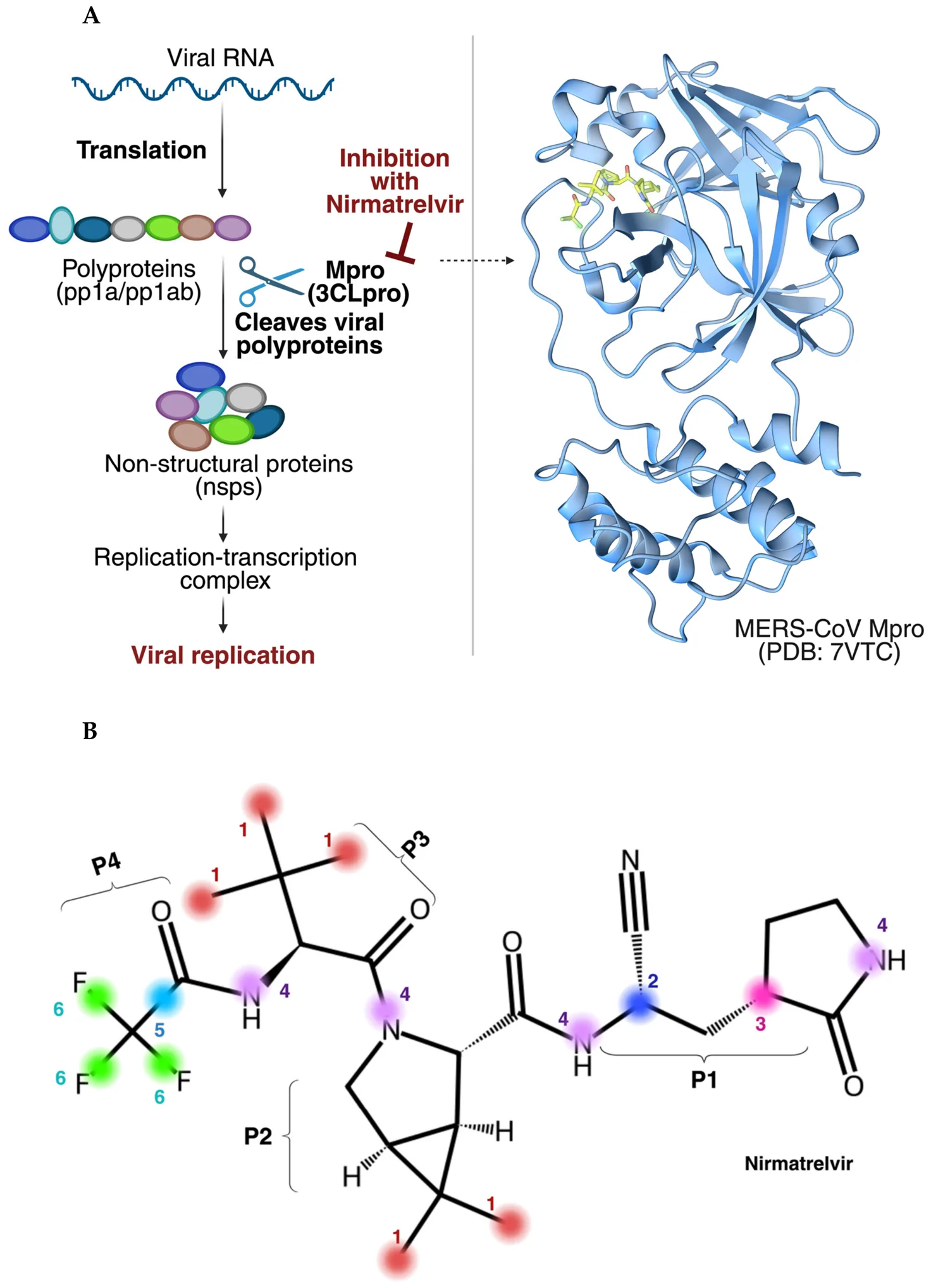
Schematic representation of the inhibition of the MERS-CoV main protease (Mpro) with Nirmatrelvir and its modifications to counter resistant mutations. (**A**) Role of MERS-CoV Mpro in viral polyprotein processing and replication, together with the crystal structure of Mpro bound to Nirmatrelvir (PDB: 7VTC). (**B**) Chemical structure of Nirmatrelvir highlighting the P1–P4 binding regions and atom annotations (highlighted) used for systematic molecular modifications.

Despite significant advances in antiviral development for SARS-CoV-2, the global community remains inadequately prepared to address a potential MERS-CoV-related outbreak, as no approved vaccine or targeted therapeutic currently exists [15]. This highlights the urgent need for enhanced surveillance, rapid diagnostic strategies, and the development of broad-spectrum antiviral agents to mitigate future coronavirus threats. Among the available therapeutic targets, the coronavirus Mpro (also known as 3CLpro) has emerged as one of the most promising due to its essential role in viral replication and its high structural conservation across betacoronaviruses. Mpro mediates the cleavage of viral polyproteins into functional nonstructural proteins, a critical step for viral maturation and propagation (Figure 1A).

Moreover, the clinical success of Nirmatrelvir (C_23_H_32_F_3_N_5_O_4_), a SARS-CoV-2 Mpro inhibitor derived from Lufotrelvir through systematic side-chain optimization [16,17], validates Mpro as a druggable therapeutic target. Notably, Nirmatrelvir also shows moderate inhibitory activity against MERS-CoV Mpro [18], indicating that rational side-chain modifications (Figure 1B) of this scaffold hold promise for developing effective inhibitors against MERS-CoV as well. However, developing such inhibitors remains a challenging and resource-intensive process. Computational approaches have therefore become essential for accelerating antiviral drug discovery, particularly for the rapid screening and prioritization of candidate compounds [19]. Among these methods, protein–ligand docking, often combined with virtual screening and molecular dynamics simulations, has emerged as a widely used technique to estimate binding affinities between ligands and their target receptors [20]. However, applying these computationally intensive methods across the vast chemical space of small molecules remains challenging due to high computational costs. In this context, recent advances in machine learning (ML) and deep learning (DL) have enabled the efficient analysis of large and complex datasets, leveraging their ability to learn intrinsic patterns and relationships within high-dimensional data spaces [11]. Several machine learning frameworks have been developed to predict protein–ligand binding affinity using different molecular and protein representations. For example, DeepDTA [21] predicts binding affinity directly from ligand SMILES strings and protein amino acid sequences using parallel one-dimensional convolutional neural networks (CNNs), whereas GraphDTA [22] represents ligands as molecular graphs processed by graph neural networks (GNNs) while encoding protein sequences with CNNs. Another deep learning framework, DEELIG [23], predicts binding affinity by learning spatial interactions between protein binding pockets and ligand features using convolutional neural networks without requiring docked ligand poses. Trained on thousands of experimentally characterized protein–ligand complexes from the RCSB Protein Data Bank (PDB) with measured binding affinities (K_d_/K_i_), DEELIG has demonstrated improved performance over conventional scoring methods on benchmark datasets such as PDBbind [24] and has also been applied to SARS-CoV-2 Mpro inhibitor complexes. Despite their strong predictive performance, these methods are designed primarily for protein targets without considering the effect of mutations in the active site. Mutation-induced changes can alter protein–ligand interactions, reduce inhibitor binding, and ultimately lead to drug resistance, particularly in rapidly evolving viral proteins. Therefore, accurate prediction of binding affinity across both wild-type and mutant proteins requires a mutation-aware framework that explicitly incorporates residue-level physicochemical changes, enabling more reliable evaluation of ligand binding and the identification of inhibitors that remain effective against emerging resistant variants.

**Figure 2:**
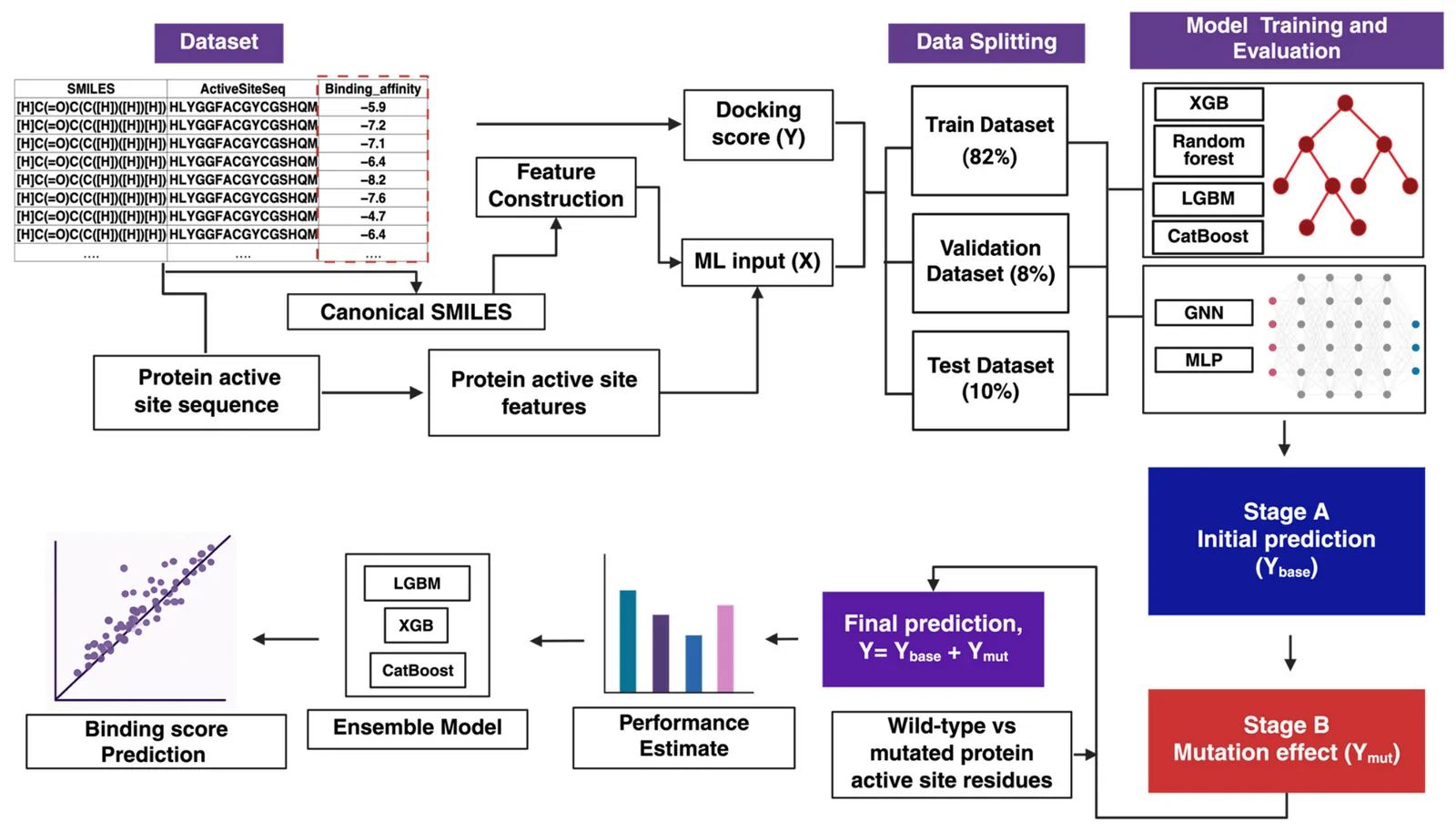
Schematic diagram of the two-stage mutation-delta modeling framework for binding-score prediction. SMILES, active-site sequences, and affinity values are processed to construct ligand and mutation features. After train/validation/test splitting, a ligand baseline model (Stage A) predicts baseline affinity (Y_base), while a mutation model (Stage B) estimates the mutation effect (Y_mut) relative to a reference sequence. The final prediction is obtained as Y = Y_base + Y_mut and evaluated using standard regression metrics.

In this study, we introduce a machine learning framework designed to predict binding affinity for Nirmatrelvir analogue molecules with Mpro and its variants to capture mutation-induced changes in protein–ligand interactions. Due to the limited availability of large-scale experimental binding affinity data for coronavirus Mpro variants, particularly across diverse mutations, we utilize docking scores as a surrogate measure of binding affinity. However, docking scores are not equivalent to experimentally determined binding affinities and provide limited representation of protein flexibility, solvent effects, water-mediated interactions, and dynamic rearrangements. Accordingly, the docking scores used here should be interpreted as computational surrogate targets, and the reported ML errors quantify prediction relative to these surrogate values rather than experimental affinity. The proposed approach decomposes docking scores into two components: (i) a ligand-specific baseline predicted from molecular descriptors, and (ii) a mutation-dependent residual term learned through delta encoding of the protein active-site sequence relative to a wild-type reference. This two-stage residual learning strategy enables the model to isolate mutation-driven perturbations while preserving ligand-specific consistency (Figure 2). By encoding only the physicochemical differences introduced by mutations, rather than treating each mutated protein as an independent entity, the framework improves interpretability and enhances generalization across mutation variants. Furthermore, ligand-grouped data splitting is employed to prevent information leakage and ensure robust model evaluation. Compared with conventional ligand-only or fully concatenated protein–ligand models, this mutation-delta approach provides a structured and mechanistically informed strategy for predicting mutation-induced changes in Mpro inhibitor binding to the protein active-site sequence relative to a wild-type reference.

## 2. Results and Discussion

### 2.1. Dataset

**Figure 3:**
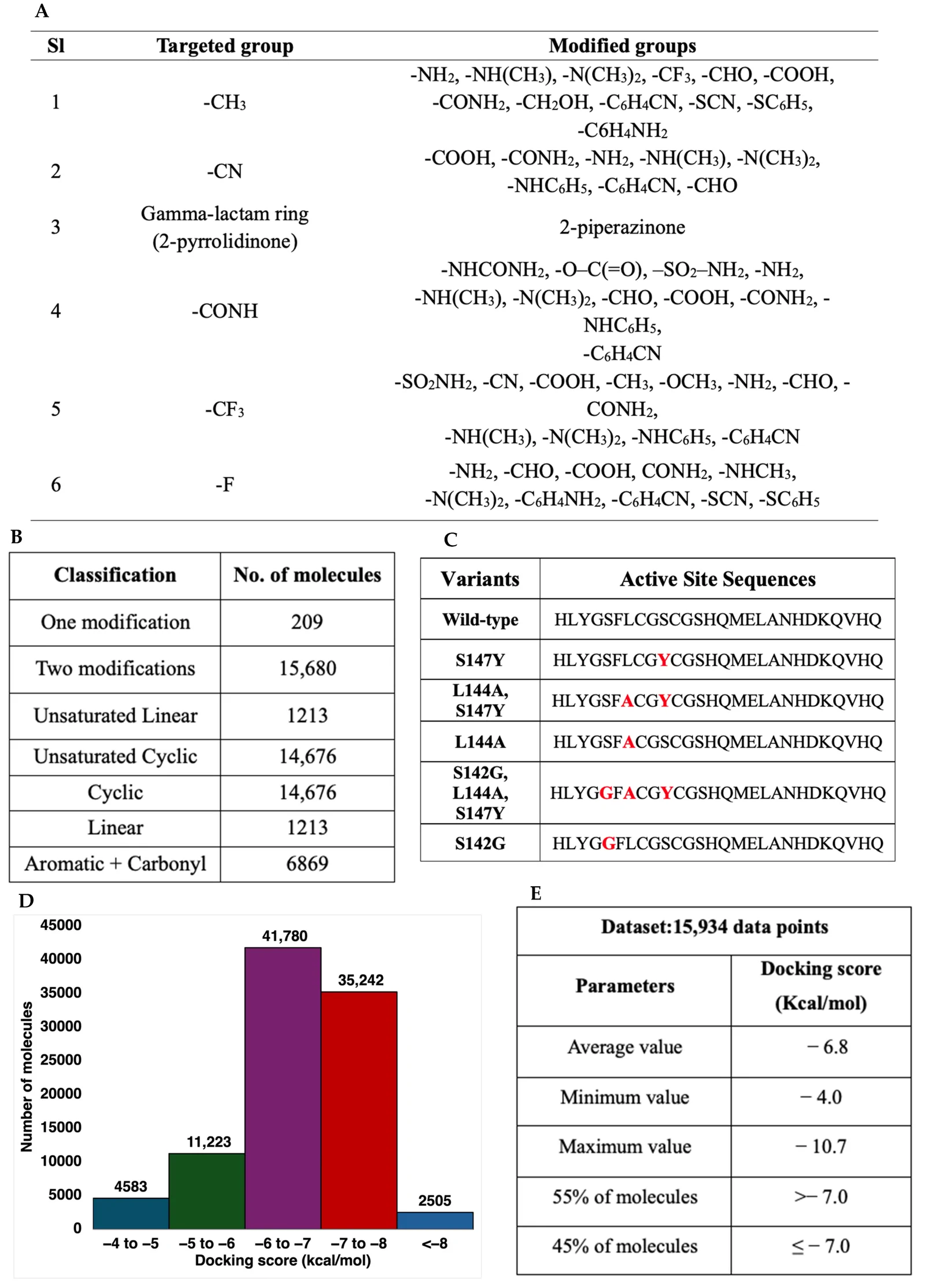
Overview of the molecular library generation, protein variants, and docking-score dataset used for machine learning model training. (**A**) Functional-group modification strategy applied to Nirmatrelvir. (**B**) Classification of the generated molecules according to structural modifications. (**C**) Active-site sequences of the wild-type and mutant MERS-CoV Mpro variants. (**D**) Distribution of docking scores across the molecular dataset. (**E**) Summary statistics of the final docking-score dataset used for model training and evaluation.

A library of 15,889 compounds was generated by systematically modifying the Nirmatrelvir scaffold at six predefined positions shown in Figure 1B using RDKit [25,26] using custom Python scripts. Molecular library generation was performed in two steps. First, a single position on the Nirmatrelvir scaffold was systematically modified using RDKit’s ReplaceSubstructs function [25] using the groups presented in Figure 3A. This yielded 209 modified molecules from the initial Nirmatrelvir structure. The resulting 209 molecules were subsequently used as starting structures for a second round of molecular generation, in which two-position modifications were introduced across all six positions using RDKit’s EnumerateLibrary function [25]. After removing duplicate and chemically invalid structures using Morgan fingerprints and canonical SMILES generated by RDKit [25], a total of 15,680 molecules were obtained (Figure 3B). All steps were automated using in-house Python scripts. The optimized structures were subsequently docked against six protein variants, including the wild-type MERS-CoV main protease (Mpro) (Figure 3C), using AutoDock Vina [27], resulting in a dataset of 95,334 protein–ligand docking complexes (Figure 3D,E).

The distribution of docking scores shows a mean docking score of −6.8 kcal/mol, with values ranging from −4.0 to −10.7 kcal/mol. A total of 45% of the compounds had docking scores ≤−7.0 kcal/mol, corresponding to stronger predicted scores, whereas 55% scored >−7.0 kcal/mol (Figure 3A). The docking score distribution further reveals that most molecules fall within the −6.0 to −7.0 kcal/mol range, followed by the −7.0 to −8.0 range (Figure 3D). Structural analysis indicates a predominance of unsaturated cyclic compounds (14,676), along with 1213 unsaturated linear molecules and 6869 compounds containing both aromatic rings and carbonyl groups (Figure 3C). Throughout this study, “linear molecules” refers to ring-truncated derivatives of Nirmatrelvir that lack its fused bicyclic and pyrrolidinone ring systems, rather than to linear molecular geometry in the strict VSEPR (Valence Shell Electron Pair Repulsion) sense. Three complementary datasets were curated to systematically investigate the properties of Nirmatrelvir analogue molecules, their interactions with the wild-type MERS-CoV main protease (Mpro), and their interactions with MERS-CoV Mpro variants.

### 2.2. Model Training with Ligand-Only Datasets

The first dataset represents a ligand-only framework, in which canonical SMILES were used to predict binding scores. The SMILES strings were converted into molecular descriptors using RDKit, including molecular weight, hydrophobicity (LogP), topological polar surface area (TPSA), hydrogen-bond donor and acceptor counts, aromaticity, and other relevant physicochemical and structural features, which served as input features for model training. The predictive performance of multiple machine learning models on a 10% test set is summarized in the Supplementary Materials Table S1. Overall, all models achieved good predictive accuracy, with ensemble methods consistently outperforming individual algorithms. The Ensemble_RF_CatBoost_LGBM model provided the best performance, achieving an R^2^ of 0.87 and a MAE of 0.21 kcal/mol, followed by Ensemble_MLP_RF_LGBM (R^2^ = 0.87, MAE = 0.21 kcal/mol). Among the individual models, LGBM, Random Forest, and CatBoost showed the highest predictive accuracy, whereas the GNN and LSTM models performed comparatively less well.

To further assess model robustness, the trained models were evaluated on an external set of 1000 previously held-out Nirmatrelvir analogues (Supplementary Materials Table S2). As expected, prediction accuracy decreased slightly compared with the internal test set, reflecting the greater structural diversity of the unseen compounds. Nevertheless, the best-performing models maintained strong predictive performance. The MLP and Random Forest models achieved the highest R^2^ (0.76) with MAE values of 0.29 and 0.30 kcal/mol, respectively, while the Ensemble_RF_CatBoost_LGBM model remained highly competitive (R^2^ = 0.75, MAE = 0.31 kcal/mol).

### 2.3. Model Training with Ligand and Active-Site Environment Datasets

Protein context was incorporated in the second dataset by integrating ligand structures with the active-site environment of MERS-CoV Mpro. Each entry includes the ligand SMILES, the 27 key active-site residues (41H, 49L, 54Y, 141G, 142S, 143F, 144L, 145C, 146G, 147S, 148C, 149G, 150S, 166H, 167Q, 168M, 169E, 170L, 171A, 172N, 175H, 190D, 191K, 192Q, 193V, 194H, 195Q), and the corresponding docking-score. Model performance on the 10% test set is presented in the Supplementary Materials Table S3. The Ensemble_RF_LGBM_CatBoost model achieved the best overall performance, with an R^2^ of 0.86 and a MAE of 0.22 kcal/mol, closely followed by CatBoost (R^2^ = 0.86, MAE = 0.22 kcal/mol). Similar to the ligand-only framework, tree-based ensemble models outperformed deep learning approaches, suggesting that the combined ligand–protein feature representation effectively captures the determinants of docking score. The generalization capability of the integrated models was subsequently evaluated using the same set of 1000 previously held-out Nirmatrelvir analogues (Supplementary Materials Table S4). Both CatBoost and the Ensemble_RF_LGBM_CatBoost model achieved the highest predictive performance, with an R^2^ of 0.77 and a MAE of 0.28 kcal/mol.

### 2.4. Model Training with Mutation-Aware Datasets

A third dataset was curated involving the active-site residues obtained from wild-type and five Mpro variants (G142, A144, Y147, A144+Y147, and G142+A144+Y147). Nirmatrelvir analogues were docked against all variants, resulting in a comprehensive dataset of 95,334 ligand–protein complexes. Each data point therefore represents a unique ligand–protein pair, defined by the ligand SMILES, the wild-type or mutated active-site sequence, and the associated docking score. This setup enables the model to learn the effects of single, double, and triple mutations on ligand binding. Of the three learning strategies investigated, the mutation-aware learning strategy was selected as the final model training framework.

### 2.5. Descriptors and Feature Generation for Mutation-Aware ML Models

The mutation-aware machine learning framework was designed to integrate the intrinsic binding characteristics of ligands with the physicochemical effects of protein mutations, enabling the model to learn the impact of sequence variations on protein–ligand interactions and reliably predict binding scores.

For each ligand, SMILES notations were converted into molecular objects using RDKit (version 2024.09.5) [28], enabling systematic descriptor calculation through the RDKit Descriptors module. A total of 217 molecular descriptors, as shown in Supplementary Materials Table S5, were initially computed for each compound. The selected descriptors encompass multiple chemically relevant categories, including constitutional descriptors (e.g., molecular weight, atom count, bond count, ring count), topological descriptors (e.g., topological polar surface area, connectivity indices, and graph-based metrics), basic electronic descriptors (e.g., partial charge-related properties), and fragment-based descriptors capturing the presence of specific functional groups and molecular substructures. Collectively, these features provide a chemically interpretable representation of molecular structure suitable for structure–property and structure–activity modeling. The graph-based model was selected from the OGB-LSC [29] leaderboard and was trained using molecular graph representations derived from the SMILES strings of the studied dataset. Molecular graphs were constructed using the OGB smiles2graph utility (version 1.3.4), which relies on the RDKit library to parse SMILES strings and generate corresponding molecular objects. From these objects, atom-level and bond-level features were extracted to form graph-based inputs for model training. In the resulting graphs, atoms are represented as nodes, with each node encoded by a set of nine features capturing atomic properties such as element type, hybridization state, chirality, and related characteristics. Bonds are represented as edges and described by three features, including bond order (single, double, triple, or aromatic), stereochemistry, and conjugation status. This graph-based representation preserves the molecular topology and chemical context required for effective GNN learning.

Protein active-site sequences were transformed into numerical descriptors using a residue-level physicochemical encoding strategy. Instead of representing amino acids as categorical symbols, each residue was mapped to a four-dimensional feature vector defined in the AA_PROPS dictionary. The selected properties were hydrophobicity (hydro), electrical charge (charge), aromaticity (arom), and polarity (polar), which are specified in scripts as PROT_KEYS = [“hydro”, “charge”, “arom”, “polar”]. Hydrophobicity values were derived from the Kyte–Doolittle hydropathy scale (1982) [30], which assigns experimentally determined hydropathy indices to amino acids based on their preference for aqueous versus nonpolar environments. In this scale, positive values correspond to hydrophobic residues (e.g., Ile = 4.5, Val = 4.2, Leu = 3.8), while negative values indicate hydrophilic residues (e.g., Arg = −4.5, Lys = −3.9, Asp = −3.5). These values were directly incorporated into the hydro feature. The charge feature encodes the net side-chain charge at physiological pH (~7.0), where positively charged residues (Lys, Arg) are assigned +1, negatively charged residues (Asp, Glu) are assigned −1, and neutral residues are assigned 0 [31]. Histidine was treated as neutral due to its partial protonation near physiological pH [32]. Aromaticity (arom) was implemented as a binary indicator, with a value of 1 assigned to residues containing aromatic side chains (Phe, Trp, Tyr, His) and 0 otherwise [31]. Similarly, polarity (polar) was encoded as a binary feature, where polar residues capable of hydrogen bonding or electrostatic interactions were assigned 1, and nonpolar residues were assigned 0. For an active-site sequence of length L, each residue was represented as a four-element vector: Residue_1_ = [hydro_1_, charge_1_, arom_1_, polar_1_].

These vectors were concatenated in sequence order, generating a fixed-length feature vector of size 4 × L. This encoding preserves residue position within the binding site while capturing key physicochemical determinants of protein–ligand recognition, including hydrophobic packing, electrostatic complementarity, π-π interactions, and hydrogen bonding potential [33]. By embedding biophysically meaningful attributes rather than categorical residue identities, the model can learn position-specific contributions of active-site residues to molecular interaction and binding specificity.

### 2.6. Feature Engineering and Descriptor Selection

Feature engineering was performed to improve model performance by selecting informative and non-redundant descriptors, thereby enhancing accuracy, reducing overfitting, and improving interpretability. Features were evaluated based on their uniqueness and correlation with the target variable to retain only relevant information and ensure model generalizability. To address multicollinearity, pairwise correlation analysis was conducted using a correlation matrix, and highly correlated features were removed based on a predefined threshold. This process eliminated 144 non-significant descriptors from an initial set of 217, resulting in 73 RDKit descriptors for the ligand-only model (Supplementary Materials Table S5). For the protein–ligand and mutation delta models, multiple descriptor sets were evaluated to determine an optimal feature subset. The final model utilized the top 20 ligand descriptors (Table 1) along with four physicochemical properties of active-site amino acid residues (Table 2), providing a balance between model performance and interpretability.

### 2.7. Model Selection

For the mutation-aware ML framework, the prediction task was divided into two stages. Stage A learned a ligand-level baseline from molecular descriptors, representing the average binding tendency of each ligand across the evaluated protein variants. Stage B learned the mutation-associated deviation from this baseline using delta-encoded physicochemical differences, in which changes in residue properties (charge, polarity, hydrophobicity, and aromaticity) between the mutant and wild-type active-site residues were used to represent each mutation. By learning only the mutation-induced changes rather than treating every protein variant as a separate target, the framework captures the effects of mutations while preserving the intrinsic binding characteristics of each ligand.

#### 2.7.1. XGBoost Regressor

**Figure 4:**
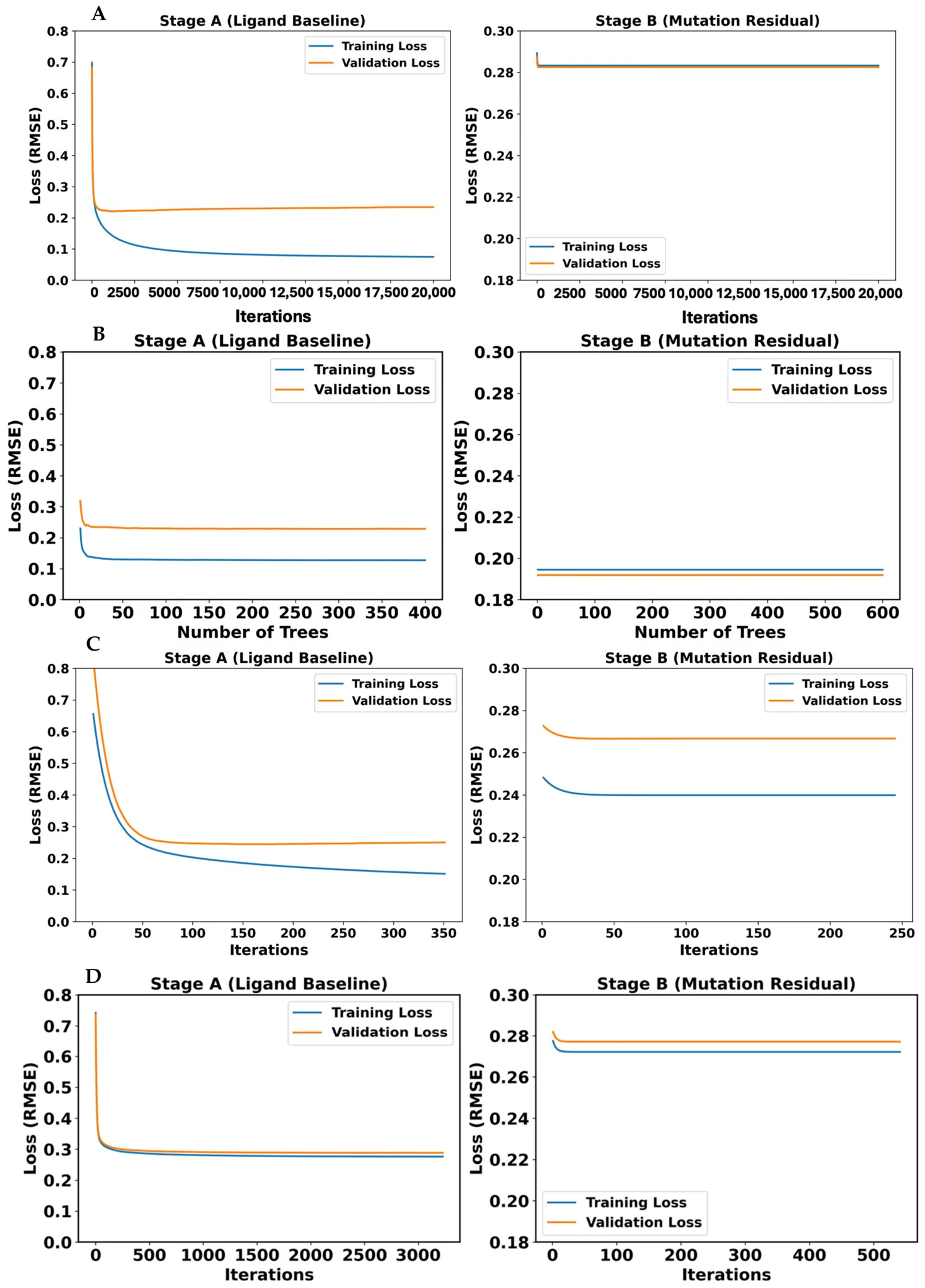
Learning curves of the mutation-aware machine learning models during training. (**A**) XGB regressor, (**B**) Random forest, (**C**) LGBM regressor, and (**D**) CatBoost regressor during the training process. The loss value is defined as the RMSE value. The learning curve of the best training session of each model are displayed.

**Figure 5:**
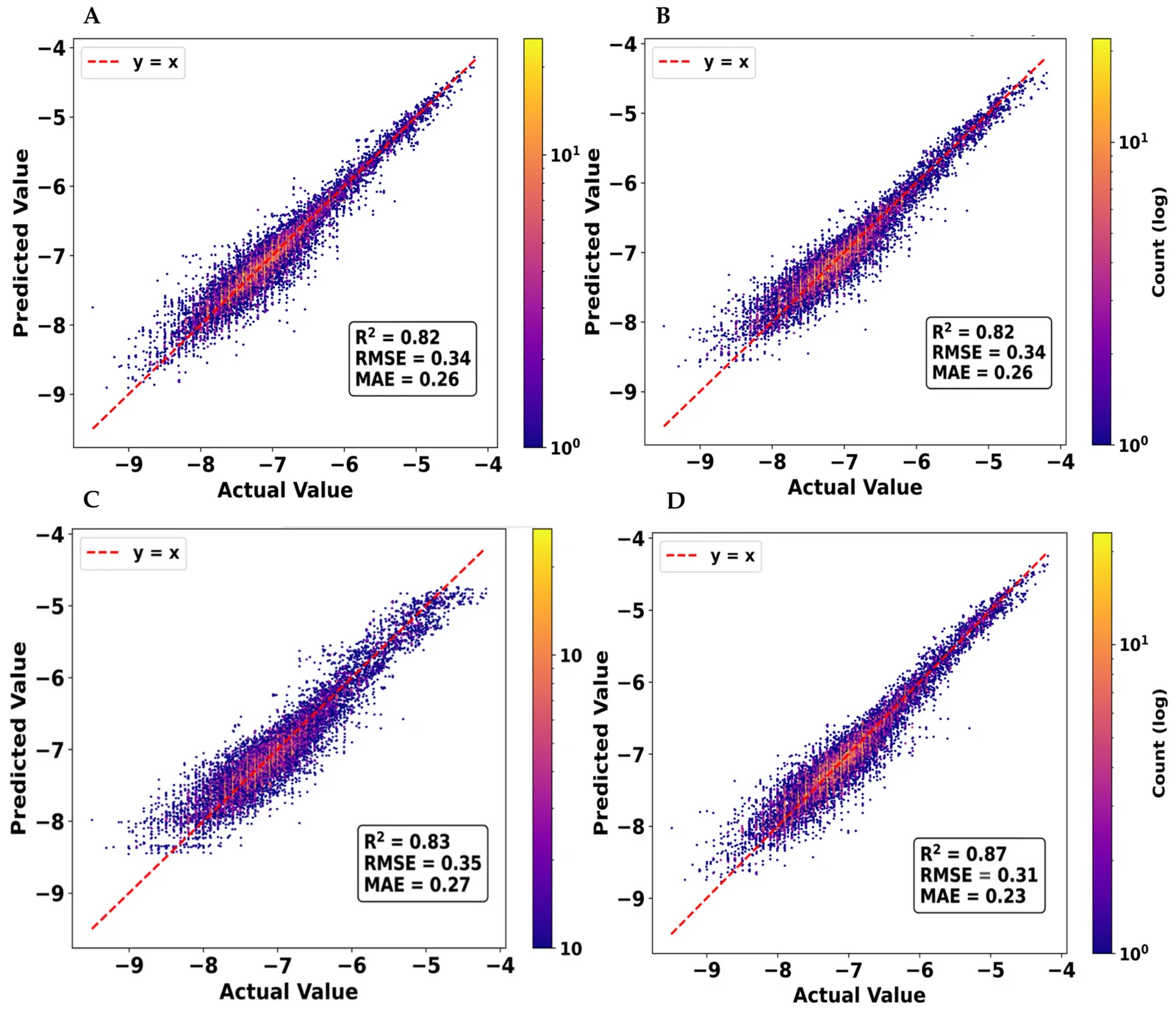
Regression plots of the optimized mutation-aware machine learning models. (**A**) XGB Regressor, (**B**) Random Forest, (**C**) LGBM regressor, and (**D**) CatBoost regressor model showing the prediction accuracy of the optimized ML models. Only the plots representing the best prediction results out of five replicate runs are shown.

XGBoost [34] is a scalable and efficient gradient boosting framework based on decision trees, widely used for handling structured and high-dimensional data such as molecular descriptors. It improves predictive performance through regularization techniques that control model complexity and reduce overfitting. XGBoost [34] employs second-order gradient optimization, parallel tree construction, and shrinkage (learning rate) to enhance both accuracy and computational efficiency. Key hyperparameters, including max_depth (4), learning_rate (0.03), subsample (0.8), colsample_bytree (0.8), min_child_weight (5), reg_alpha (0.0), and reg_lambda (2.0), were optimized. The model was trained with a maximum of 20,000 boosting rounds. Performance was monitored using root mean squared error (RMSE) to ensure generalization and prevent overfitting. A 5-fold cross-validation strategy was employed. Five replicate training and validation runs were conducted using different random seeds (1001, 1002, 1003, 1004, and 1005) while maintaining identical data splits to ensure robustness and reproducibility (Supplementary Materials Table S6). The best-performing fold (Fold 2) achieved an R^2^ of 0.82, RMSE of 0.34, and MAE of 0.26 for the mutation delta model (Figure 4A and Figure 5A).

#### 2.7.2. Random Forest Regressor

Random Forest [35] is an ensemble learning method based on multiple decision trees, designed to improve predictive accuracy and reduce overfitting through bootstrap aggregation (bagging). It is particularly effective for handling high-dimensional molecular descriptor data due to its ability to capture non-linear relationships and interactions between features. By averaging predictions across multiple trees, Random Forest enhances model robustness and stability while reducing variance. Key hyperparameters, including n_estimators (500), max_depth (20), max_features (sqrt), min_samples_split (5), and min_samples_leaf (2), were optimized to achieve the best performance. A 5-fold cross-validation strategy was employed to ensure reliable model evaluation and generalization. Five replicate training and validation runs were performed using different random seeds (1001, 1002, 1003, 1004, and 1005) while maintaining identical data splits to ensure robustness and reproducibility (Supplementary Materials Table S6). The best-performing fold (Fold 4) achieved an R^2^ of 0.82, RMSE of 0.34, and MAE of 0.26 (Figure 4B and Figure 5B). Across all folds, the model demonstrated consistent performance with minimal variation, indicating stable learning behavior and reliable predictive capability.

#### 2.7.3. LGBM Regressor

LightGBM [36] is a fast, distributed, and efficient gradient boosting framework suitable for handling high-dimensional molecular descriptor data. It is based on decision trees and incorporates advanced techniques such as Gradient-based One-Side Sampling (GOSS) and histogram-based learning to improve computational efficiency and reduce training time. Its ability to handle large feature spaces and provide feature importance makes it well-suited for understanding descriptor contributions to model predictions. Key hyperparameters, including num_leaves (63), learning_rate (0.05), max_depth (−1), min_child_samples (50), subsample (0.8), colsample_bytree (0.8), reg_alpha (0.001), and reg_lambda (1.0), were optimized. The model was trained with up to 20,000 boosting iterations, with early stopping applied after 200 rounds without improvement on the validation set. Model performance was monitored using mean squared error (MSE) to ensure generalization and prevent overfitting. A 5-fold cross-validation strategy was employed. Five replicate training and validation runs were conducted using different random seeds (1001, 1002, 1003, 1004, and 1005) while maintaining identical data splits to ensure robustness and reproducibility (Supplementary Materials Table S6). The best-performing fold (Fold 3) achieved an R^2^ of 0.83, RMSE of 0.35, and MAE of 0.27 (Figure 4C and Figure 5C).

#### 2.7.4. CatBoost Regressor

CatBoost [37] is a gradient boosting framework based on symmetric decision trees, designed to improve prediction accuracy while reducing overfitting. It incorporates ordered boosting and advanced regularization techniques, enabling efficient handling of complex, high-dimensional datasets such as molecular descriptors. CatBoost is particularly effective in capturing non-linear relationships and feature interactions while maintaining stability across training iterations. Key hyperparameters, including depth (6), learning_rate (0.1), iterations (4000), l2_leaf_reg (10), bagging_temperature (0.0), and random_strength (1.0), were optimized. The model was trained using an iterative boosting approach, with performance monitored on the validation set to prevent overfitting and ensure generalization. A 5-fold cross-validation strategy was employed. Five replicate training and validation runs were conducted using different random seeds (1001, 1002, 1003, 1004, and 1005) while maintaining identical data splits to ensure robustness and reproducibility (Supplementary Materials Table S6). The best-performing fold (Fold 3) achieved an R^2^ of 0.87, RMSE of 0.31, and MAE of 0.23 (Figure 4D and Figure 5D). Across all folds, the model demonstrated consistent performance with minimal variation, indicating stable learning behavior and reliable predictive capability.

#### 2.7.5. Graph Neural Network (GNN)

A GNN [38] was employed to capture the structural information of ligands by representing each molecule as a graph, where atoms correspond to nodes and chemical bonds to edges. This representation enables the model to learn spatial and topological relationships directly, making it well-suited for molecular property prediction tasks. The GNN model aggregates information from neighboring atoms through iterative message-passing operations, allowing the extraction of rich, context-aware features that reflect the molecular structure. The baseline GNN architecture utilized a hidden dimension of 128 and was trained using a learning rate of 0.0005 for up to 300 epochs. A 5-fold cross-validation strategy was employed. Five replicate training and validation runs were conducted using different random seeds (1001, 1002, 1003, 1004, and 1005) while maintaining identical data splits to ensure robustness and reproducibility (Supplementary Materials Table S6). The best-performing fold (Fold 4) achieved an R^2^ of 0.78, RMSE of 0.38, and MAE of 0.30.

#### 2.7.6. MLP Regressor

MLP [39] is a fully connected feedforward neural network and one of the foundational architectures in deep learning. It consists of an input layer, multiple hidden layers with non-linear activation functions, and an output layer. MLP models are well-suited for capturing complex non-linear relationships in high-dimensional molecular descriptor data. In this study, RDKit descriptors were first scaled using a standard scaler and used as input features for training the model. The fully connected architecture enables the model to learn intricate interactions among molecular features. Key hyperparameters, including hidden_layer_sizes ([256, 256, 128, 128, 64, 64, 32, 32, 16]), activation function (‘relu’), solver (‘adam’), learning rate (‘adaptive’), and regularization parameter alpha (0.005), were optimized to achieve the best model performance. A 5-fold cross-validation strategy was employed to ensure robustness and generalization. Five replicate training runs were performed using different random seeds (1001, 1002, 1003, 1004, and 1005) while maintaining identical data splits (Supplementary Materials Table S6). The prediction loss (RMSE) on the validation dataset reached its minimum at approximately 127 epochs for the best-performing folds. The best-performing fold (Fold 3) achieved an R^2^ of 0.84, RMSE of 0.35, and MAE of 0.27.

### 2.8. Ensemble Model

**Figure 6:**
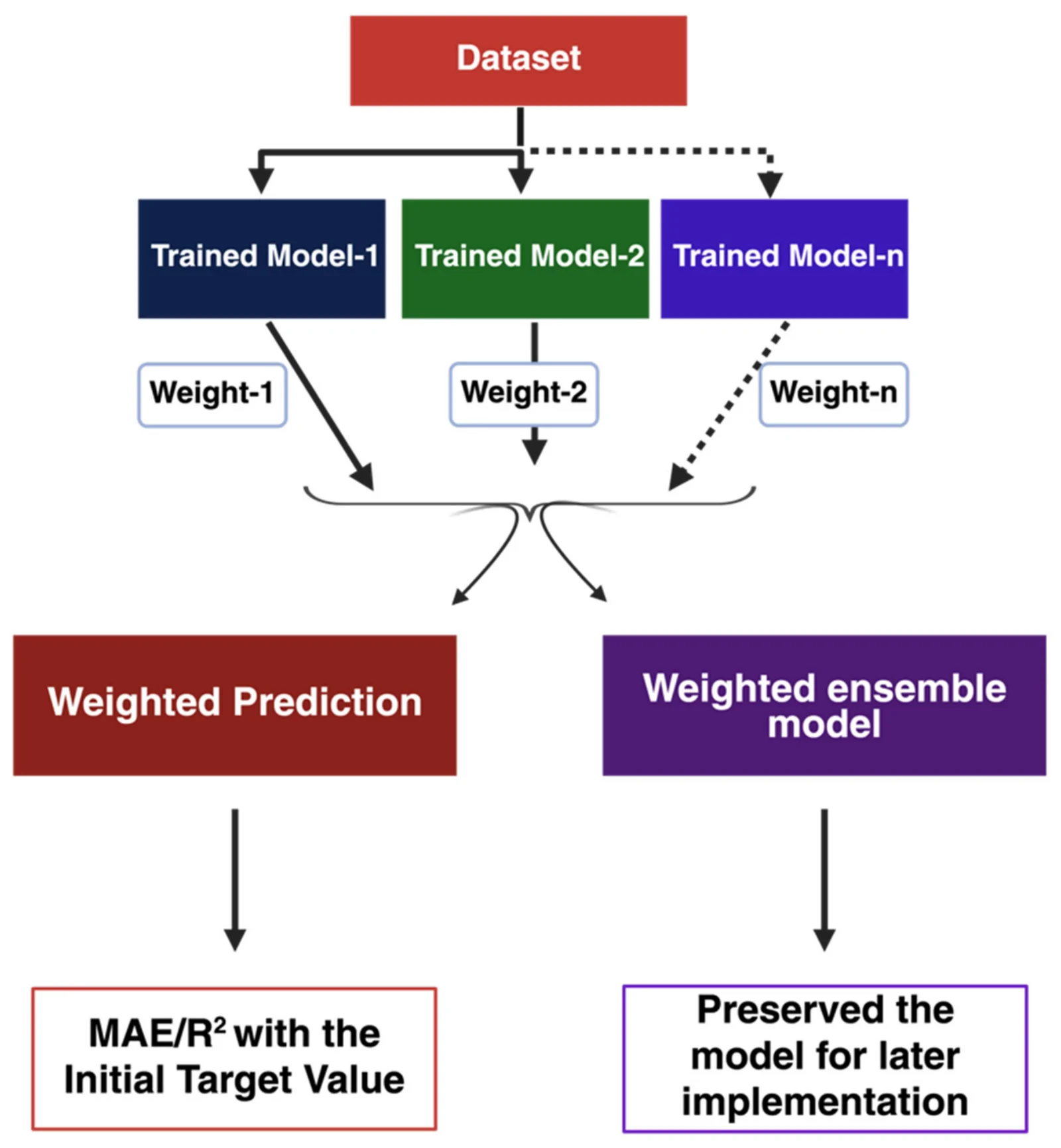
Schematic representation of the weighted ensemble modeling framework. The dataset is used to train multiple independent models, each generating predictions with assigned weights. These weighted outputs are combined to produce a final ensemble prediction, which is evaluated using performance metrics such as MAE and R^2^ against the initial target values. The resulting weighted ensemble model is preserved for subsequent deployment and implementation.

The final ensemble model was constructed using a systematic weight-grid approach (Figure 6) integrating CatBoost, XGBoost, and LightGBM models. For the ensemble model, predictions were generated as the sum of two components: a ligand-based baseline prediction and a mutation-driven residual correction, ensuring incorporation of both molecular and protein sequence information. These predictions were combined using weighted averaging, where weights were varied from 0.0 to 1.0 in increments of 0.1, constrained to sum to 1.0, generating 66 ensemble combinations (Supplementary Materials Table S7). Model evaluation was performed using a ligand-grouped 5-fold cross-validation strategy (GroupKFold) to compute out-of-fold (OOF) metrics, along with a fixed 10% ligand-grouped test split to ensure unbiased performance assessment. For each weight combination, ensemble predictions were evaluated using R^2^, RMSE, and MAE on both OOF and test datasets. The optimal ensemble configuration was selected based on the lowest test MAE and R^2^. The final model was saved as a self-contained bundle, including trained base models, mutation models, descriptor configurations, and optimal weights, enabling reproducible predictions. Additionally, prediction outputs were decomposed into baseline (ligand) and residual (mutation) contributions.

**Figure 7:**
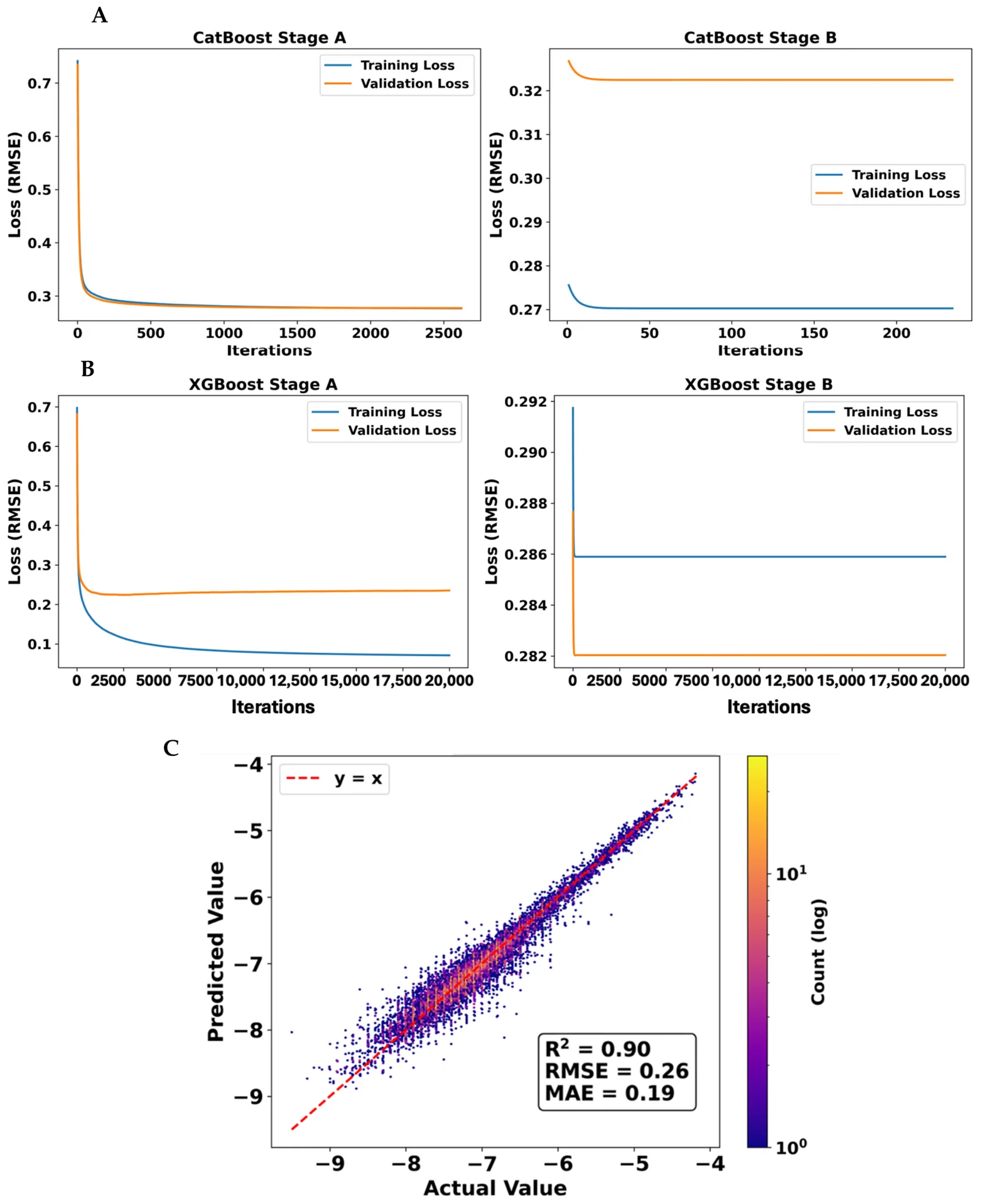
Performance of the ensemble model combining CatBoost and XGBoost. (**A**) Training and validation loss curves for CatBoost (Stage A and Stage B) and (**B**) XGBoost (Stage A and Stage B) models demonstrate convergence and stability during training. (**C**) The ensemble model predictions are evaluated using R^2^ = 0.90, RMSE = 0.26, and MAE = 0.19, indicating high predictive accuracy and reliability.

Using this approach, a weighted ensemble of CatBoost, XGBoost, and LightGBM was constructed. The optimal ensemble assigned weights of 0.8 to CatBoost, 0.1 to XGBoost, and 0.1 to LightGBM (Supplementary Materials Table S7), indicating that the final prediction was derived solely from the CatBoost and XGBoost models. This ensemble achieved a test MAE of 0.19, RMSE of 0.26, and R^2^ of 0.90, demonstrating stable and reliable predictive performance (Figure 7). These results indicate that the weighted ensemble strategy improved prediction accuracy and robustness compared with the individual models.

### 2.9. Evaluation of Ligand-Mutation Interaction-Aware Modeling

The original framework captures mutation-associated variation through its two-stage design. Stage A establishes a ligand-level baseline based on the average binding tendency of each ligand across all evaluated protein variants, thereby incorporating the overall binding behavior observed across the variant space, while Stage B uses delta-encoded physicochemical changes to model deviations associated with individual mutations. However, because Stage B does not include ligand features, the mutation-associated correction for a given variant is not explicitly ligand-dependent. To address this limitation, an interaction-aware Stage B was developed by jointly incorporating ligand and mutation features (Section 3.2). The interaction-aware weighted ensemble achieved an of 0.87 and a MAE of 0.20 kcal/mol (Supplementary Materials Table S8), compared with an of 0.90 and a MAE of 0.19 kcal/mol for the original framework. Although the interaction-aware formulation enables explicit ligand-specific mutation responses, it did not improve overall predictive performance for the present dataset. Therefore, the original mutation-aware framework was retained as the primary model.

### 2.10. Comparison of ML Models Trained with Molecular Graph and Descriptors

**Figure 8:**
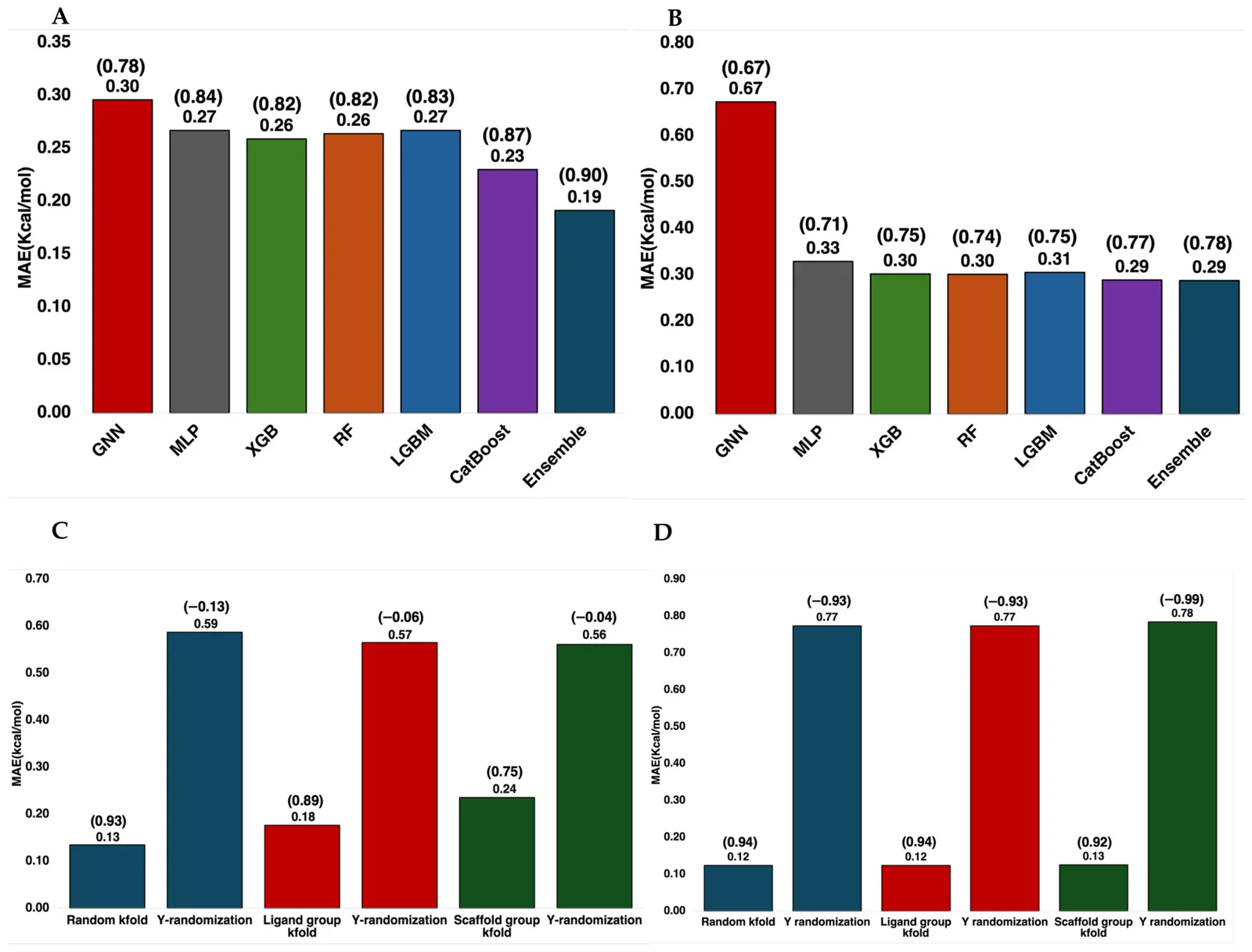
Comparative evaluation and validation of machine learning models for binding-affinity prediction. (**A**) Performance comparison of GNN, MLP, XGBoost (XGB), Random Forest (RF), LightGBM (LGBM), CatBoost, and an ensemble model (CatBoost + XGB + LGBM) on the 10% test dataset, evaluated using mean absolute error (MAE), with the corresponding R^2^ shown in parentheses. (**B**) Performance of the same models on an independent test set of 1000 molecules. The ensemble model achieved the highest predictive accuracy, exhibiting the lowest MAE and highest R^2^ among all evaluated models. (**C**) Robustness assessment of the CatBoost model using random k-fold, ligand-group k-fold, scaffold-group k-fold cross-validation, and the corresponding Y-randomization tests. (**D**) Robustness assessment of the ensemble model using the same validation strategies. The substantial increase in prediction error and decrease in R^2^ following Y-randomization confirm that the models capture meaningful structure–activity relationships rather than random correlations, demonstrating good generalization and resistance to overfitting.

Among the three modeling strategies, the mutation-delta approach demonstrated strong predictive performance on the held-out test set. On the 10% test data, tree-based models consistently outperformed neural network-based approaches. CatBoost achieved the best performance with a MAE of 0.23 Kcal/mol and R^2^ of 0.87, followed by LightGBM (MAE = 0.27 Kcal/mol, R^2^ = 0.83) and XGBoost (MAE = 0.26 Kcal/mol, R^2^ = 0.82), while Random Forest showed comparable performance (MAE = 0.26 Kcal/mol, R^2^ = 0.82). Neural network-based models (MLP) achieved a MAE of 0.27 Kcal/mol (R^2^ = 0.84). In contrast, the GNN showed the lowest performance (MAE = 0.30 Kcal/mol, R^2^ = 0.78) (Figure 8A). The mutation-delta framework enabled all models to generate decomposed predictions, explicitly separating ligand baseline contributions from mutation-driven effects. This decomposition provided direct insight into how specific protein mutations influence docking-score relative to a given ligand.

Notably, ensemble approaches delivered the highest overall performance. The ensemble model achieved the lowest error (MAE = 0.19 Kcal/mol, R^2^ = 0.90), outperforming all individual models. This improvement highlights that combining tree-based models enhances predictive stability and reduces variance, particularly when applied to unseen data.

For additional evaluation, all models were applied to a set of 1000 newly generated Nirmatrelvir analogues that were excluded from the training, validation, and test datasets. Importantly, these compounds were generated using functional-group modifications distinct from those used to construct the original dataset, thereby introducing previously unseen substitutions around the Nirmatrelvir scaffold (Supplementary Materials Table S9). Although the compounds retain the common Nirmatrelvir core and therefore do not represent a completely unrelated chemical space, this evaluation provides a more stringent assessment of model generalization toward novel functional-group modifications within the Nirmatrelvir-centered chemical space. Tree-based models again outperformed neural network approaches. CatBoost and ensemble model (CatBoost + XGB + LGBM) achieved the best performance with a MAE of 0.289 Kcal/mol, R^2^ = 0.77, and MAE of 0.29 Kcal/mol, R^2^ = 0.78 respectively, followed closely by LightGBM (MAE = 0.30 Kcal/mol, R^2^ = 0.75), XGBoost and Random Forest (MAE = 0.30 Kcal/mol, R^2^ = 0.75 and MAE of 0.30 Kcal/mol, R^2^ = 0.74 respectively). The MLP showed moderate performance (MAE = 0.33 Kcal/mol, R^2^ = 0.71), while the GNN exhibited the lowest accuracy (MAE = 0.67 Kcal/mol, R^2^ = 0.67) (Figure 8B). Overall, the ensemble model provided the most robust predictions, demonstrating improved stability and generalization compared to individual models.

The two best-performing models were evaluated using the R^2^ and MAE under three cross-validation strategies. Random repeated 5-fold cross-validation (5 repetitions; 25 train-test splits), 5-fold ligand-based GroupKFold, and 5-fold Murcko scaffold-based GroupKFold (Supplementary Materials Tables S10 and S11). The repeated random K-fold approach provided a robust baseline estimate of predictive performance across multiple random data partitions, whereas the ligand- and scaffold-based GroupKFold strategies assessed the model’s ability to generalize to previously unseen ligands and novel chemical scaffolds, respectively. To further verify model robustness and exclude the possibility of chance correlations, Y-randomization (response permutation) was performed by randomly shuffling the docking score labels while keeping the molecular descriptors unchanged. This label-shuffling procedure was repeated 20 independent times for each cross-validation strategy, and the models were retrained and evaluated after each shuffle. The substantial decrease in predictive performance following Y-randomization confirmed that the models captured genuine feature–target relationships rather than fitting random noise, demonstrating that the observed predictive accuracy was not due to chance. However, Y-randomization does not establish generalization to mutations or chemical structures absent from the training data.

The CatBoost model achieved strong predictive performance under repeated random 5-fold cross-validation (R^2^ = 0.93, MAE = 0.14 Kcal/mol) and ligand-based GroupKFold (R^2^ = 0.89, MAE = 0.18 Kcal/mol). However, its performance declined under scaffold-based GroupKFold (R^2^ = 0.75, MAE = 0.24 Kcal/mol), suggesting reduced generalization to compounds with previously unseen chemical scaffolds (Figure 8C). In contrast, the ensemble model (CatBoost + XGBoost + LGBM) consistently outperformed the individual model across all validation strategies, achieving R^2^ values of 0.94, 0.94, and 0.92, with corresponding MAE values of 0.12, 0.12, and 0.13 Kcal/mol for repeated random 5-fold, ligand-based GroupKFold, and scaffold-based GroupKFold, respectively (Figure 8D). Following Y-randomization, all validation strategies produced negative R^2^ values (−0.13 to −0.99) and substantially higher MAE values (0.57–0.77 Kcal/mol), demonstrating that the models learned genuine relationships between molecular features and docking scores rather than fitting random noise.

To assess variability in the docking-derived values, 1235 representative molecules (approximately 8% of total generated molecules) were independently docked with MERS-CoV Mpro five times under identical docking parameters, with independently generated random seeds. The standard deviation (SD) of the docking scores from five independent runs was calculated separately for each molecule, and the average SD across the 1235 molecules was 0.16 kcal/mol (median: 0.10; range: 0.0–1.0 kcal/mol) (Supplementary Materials, Replicate docking score.csv). This variability of the docking scores is comparable to the ensemble MAE of 0.19 kcal/mol, indicating similarity between model prediction error and the variability associated with the docking score.

### 2.11. Model Performance Across Molecular Structural Classes

**Figure 9:**
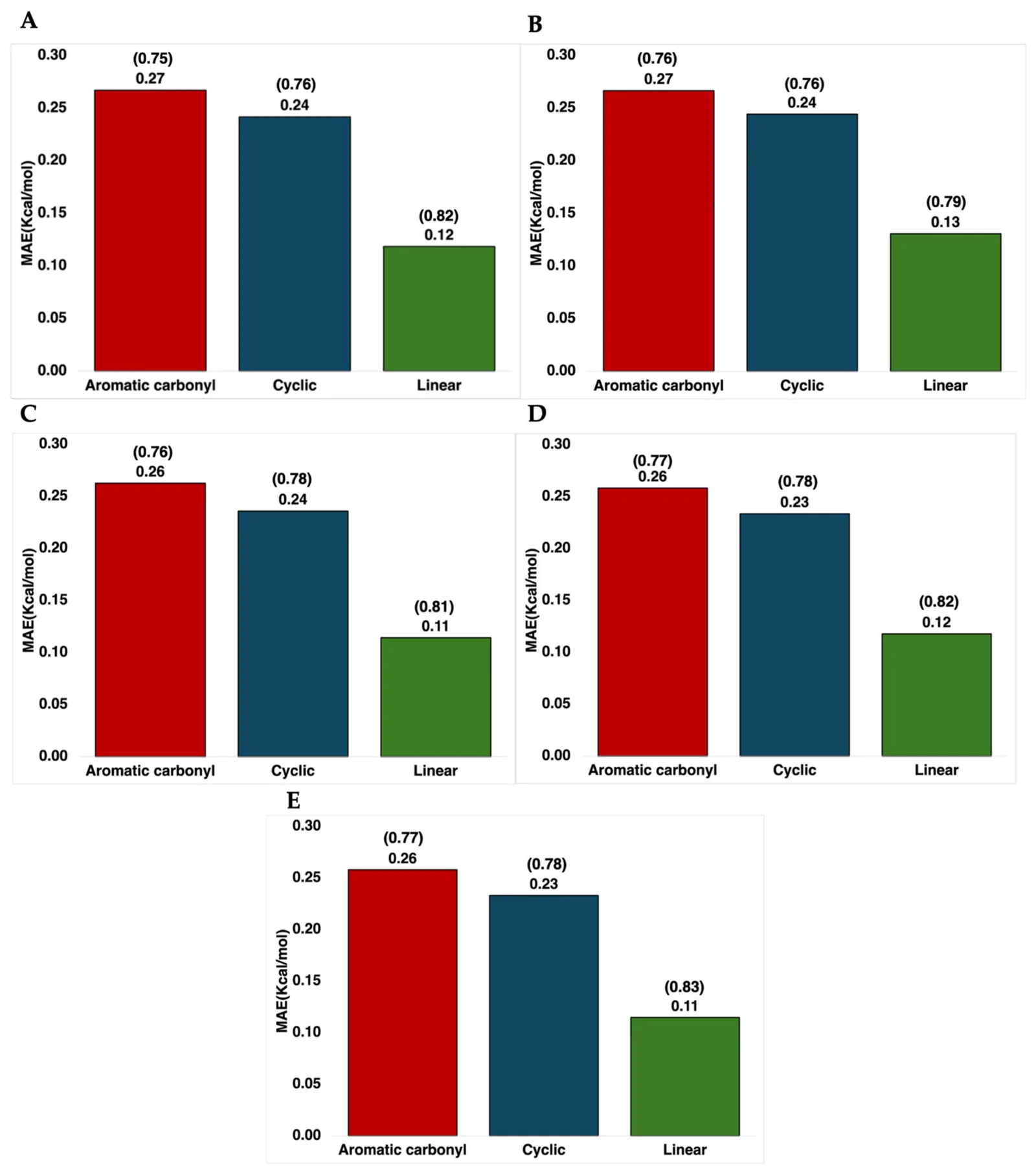
Comparison of model performance across different structural classes (aromatic carbonyl, cyclic, and linear) using five machine learning approaches: (**A**) XGBoost (XGB), (**B**) Random Forest (RF), (**C**) LightGBM (LGBM), (**D**) CatBoost, and (**E**) Ensemble model.

The performance of different machine learning models was evaluated across different chemical scaffolds; the test set was categorized into three structural classes: aromatic carbonyl, cyclic, and linear (Figure 9). Across all five machine learning models, the linear compounds consistently exhibited the highest predictive accuracy, whereas the aromatic carbonyl compounds were the most challenging to predict. For the aromatic carbonyl class, XGBoost achieved a MAE of 0.27 kcal/mol with an R^2^ of 0.76, the cyclic class showed a MAE of 0.24 kcal/mol with an R^2^ of 0.77, and the linear class produced the lowest MAE (0.12 kcal/mol) and the highest R^2^ (0.82) (Figure 9A). A similar trend was observed for Random Forest (Figure 9B), with MAE/R^2^ values of 0.27/0.77, 0.24/0.76, and 0.13/0.80 for the aromatic carbonyl, cyclic, and linear classes, respectively. LightGBM (Figure 9C) yielded MAE/R^2^ values of 0.26/0.77 for aromatic carbonyl compounds, 0.24/0.79 for cyclic compounds, and 0.11/0.81 for linear compounds. CatBoost (Figure 9D) achieved MAE/R^2^ values of 0.26/0.77, 0.23/0.78, and 0.12/0.82 for the aromatic carbonyl, cyclic, and linear classes, respectively. The ensemble model (Figure 9E) demonstrated the best overall performance, with MAE/R^2^ values of 0.26/0.77 for aromatic carbonyl compounds, 0.23/0.78 for cyclic compounds, and 0.11/0.83 for linear compounds.

### 2.12. SHAP Analysis

**Figure 10:**
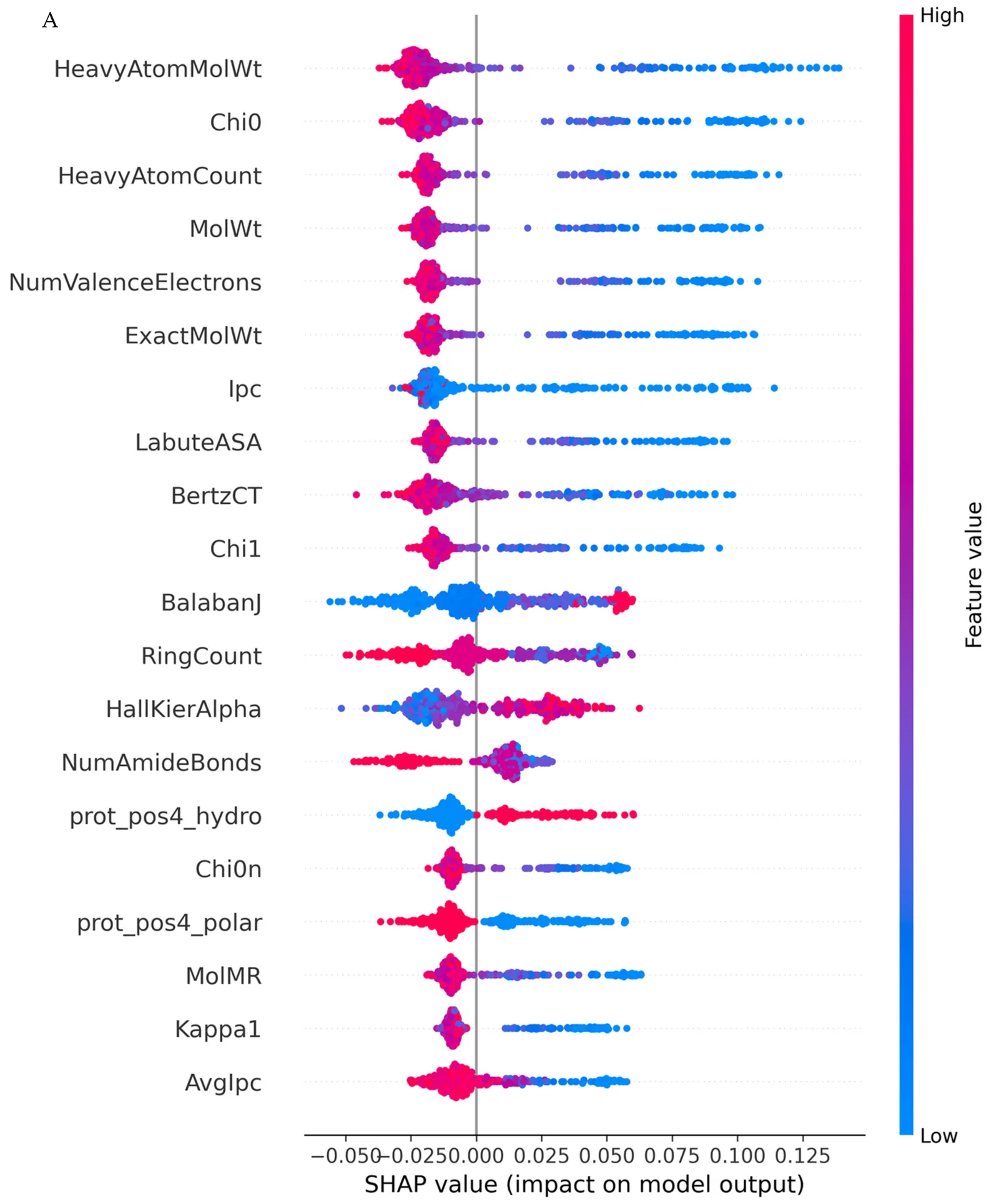

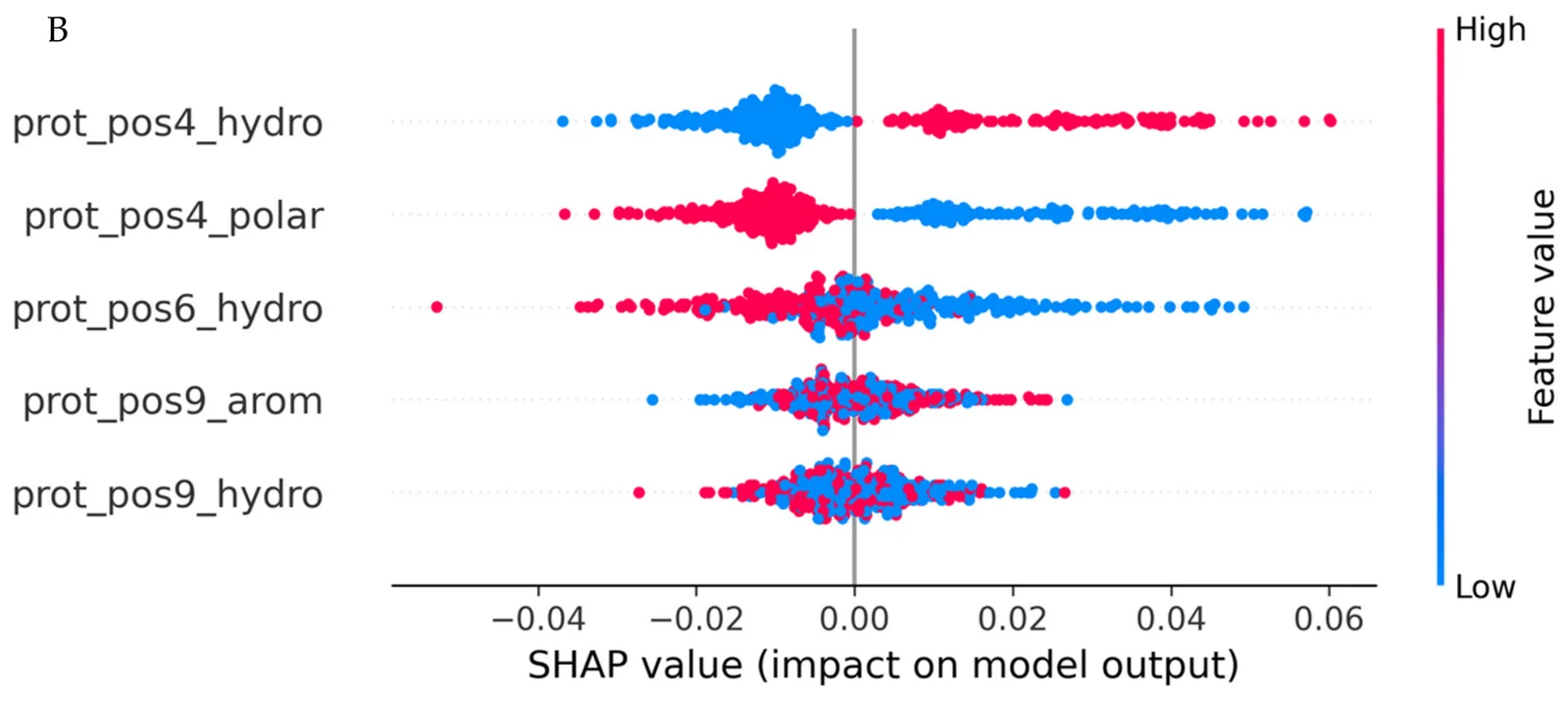
(**A**) Top 20 most important features for ligand and (**B**) Top 5 most important protein active-site features were identified through SHAP analysis by RF regressor model for predicting the docking score.

SHapley Additive exPlanations (SHAP) analysis was performed using the best-performing individual model, the CatBoost regressor, to evaluate feature importance and quantify the contribution of individual molecular descriptors to binding score predictions. The impact of the top 20 features on the predicted scores is illustrated in Figure 10, providing insights into how specific molecular and protein features influence docking scores.

The top 20 features identified by the CatBoost regressor for binding-affinity prediction are presented in Figure 10A, while key protein active-site features are highlighted in Figure 10B. In both figures, red dots represent higher feature values, whereas blue dots indicate lower feature values. Dots positioned on the left side of the vertical axis correspond to a negative contribution (stronger binding), while those on the right indicate a positive contribution (weaker binding). The spread and overlap of points reflect the variability and interaction of features in influencing model predictions.

From the SHAP analysis, molecular descriptors such as HeavyAtomMolWt, HeavyAtomCount, MolWt, NumValenceElectrons, and ExactMolWt show the largest impact on docking score prediction. These features primarily capture molecular size, atomic composition, and electronic structure, which are critical determinants of docking score. The SHAP distribution indicates that larger and heavier molecules (higher values of these descriptors) generally contribute toward stronger binding (more negative binding score), likely due to increased intermolecular interactions within the binding pocket. Similarly, descriptors such as BertzCT, HallKierAlpha, and RingCount reflect molecular complexity, branching, and cyclicity, where increased structural complexity tends to enhance binding interactions. Features like LabuteASA and Ipc, which describe surface area and topological information, further indicate that molecular size and accessible surface area play significant roles in stabilizing ligand–protein interactions. (Table 1).

In addition to ligand descriptors, protein-derived features such as prot_pos4_hydro, prot_pos4_polar, prot_pos6_hydro, prot_pos9_arom, and prot_pos9_hydro (Table 2) significantly influence binding affinities. These features represent physicochemical changes at specific active-site positions. The SHAP analysis suggests that increased hydrophobicity at certain positions (e.g., prot_pos4_hydro) enhances the docking score, while higher polarity (prot_pos4_polar) can either stabilize or destabilize interactions depending on ligand compatibility. Aromatic and hydrophobic contributions at positions such as prot_pos9 also play a crucial role, highlighting the importance of local residue environment in modulating ligand binding (Table 2).

**Figure 11:**
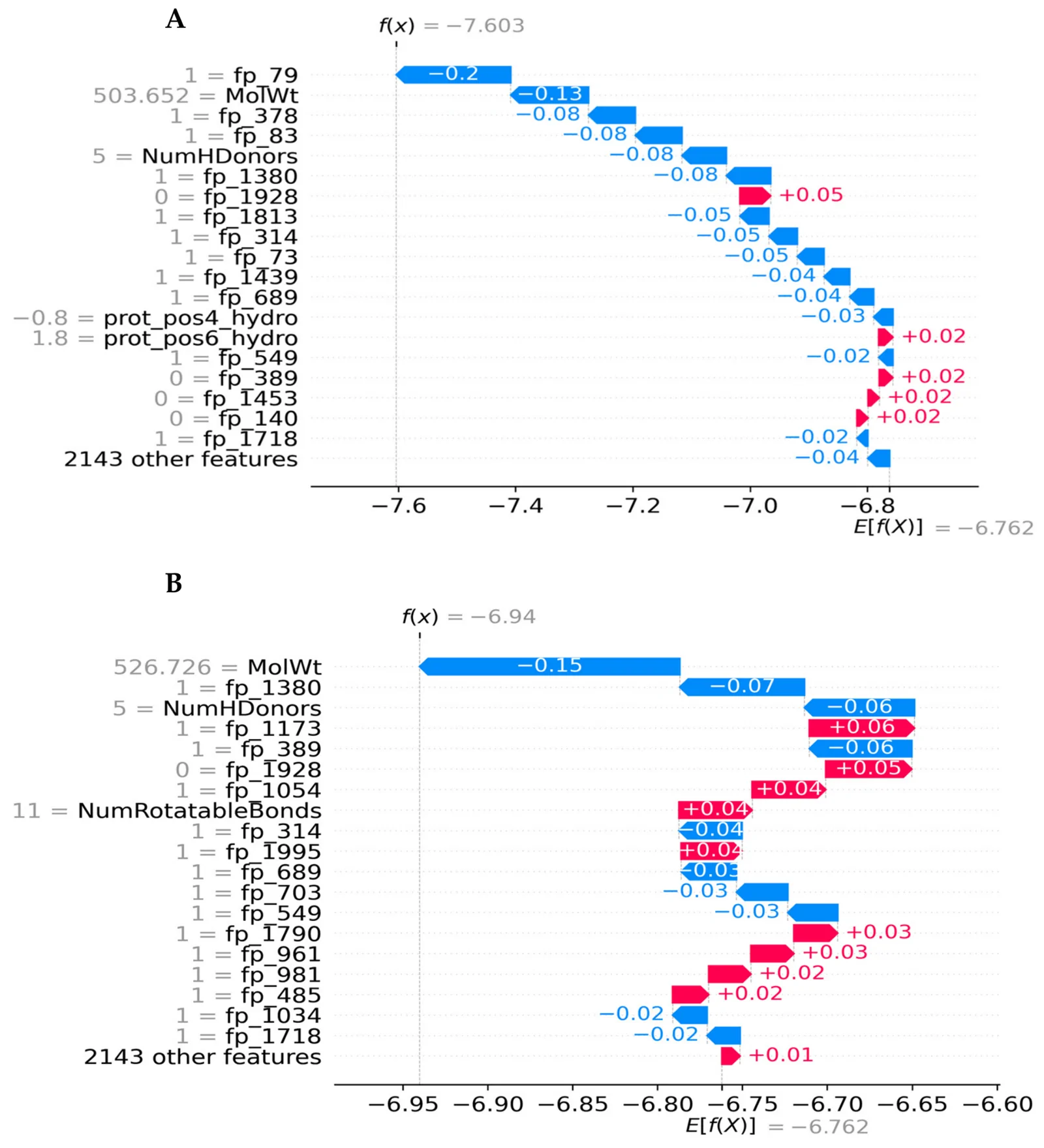
Waterfall plot illustrating the contributions of important features for a single molecule obtained from (**A**) the CatBoost regressor model, and (**B**) the Ensemble model.

The prediction of the binding score for a representative molecule by the CatBoost regressor is illustrated in Figure 11A, where the contribution of individual features is analyzed. The x-axis represents the predicted docking score, with E[f(x)] denoting the baseline (expected value), while the final model prediction for the molecule is f(x). The values shown to the left of each feature correspond to the actual input values for that molecule. Within the plot, red bars indicate features that increase the predicted docking score (weaker binding), whereas blue bars represent features that decrease the docking score (stronger binding).

Key molecular descriptors such as MolWt, NumHDonors, and fp-based structural features (e.g., fp_79, fp_378, fp_83) show significant contributions. For instance, molecular weight and structural fingerprint features contribute negatively, indicating an enhanced docking score (lower docking score), while certain fingerprint features and electronic descriptors contribute positively, slightly weakening binding. Additionally, protein-derived features such as prot_pos4_hydro and prot_pos4_polar demonstrate noticeable influence, highlighting the role of local active-site environment in modulating ligand binding. The combined contribution of numerous minor features further refines the final prediction.

Similarly, the ensemble model prediction for the same molecule is shown in Figure 11B. The baseline value shifts slightly, with the final prediction reflecting contributions from both ligand and protein features. Molecular descriptors such as MolWt and NumHDonors again play a dominant role in decreasing the docking score, indicating stronger binding, while features such as NumRotatableBonds and specific fingerprint descriptors (e.g., fp_1380, fp_173) contribute positively, slightly increasing the docking score. The cumulative effect of all features results in the final predicted docking score.

Overall, the SHAP-based analysis confirms that docking score predictions are driven by a combination of molecular size, structural fingerprints, hydrogen bonding capacity, and protein active-site properties. These findings are consistent with known principles of protein–ligand interactions, reinforcing the interpretability and reliability of the model. However, additional descriptors, including residue volume, hydrogen-bonding capacity, solvent accessibility, side-chain flexibility, and evolutionary conservation, can be incorporated to further improve the representation of mutation effects.

### 2.13. Evaluation of Mutation Sensitivity of the Final Model and Its Applicability

**Figure 12:**
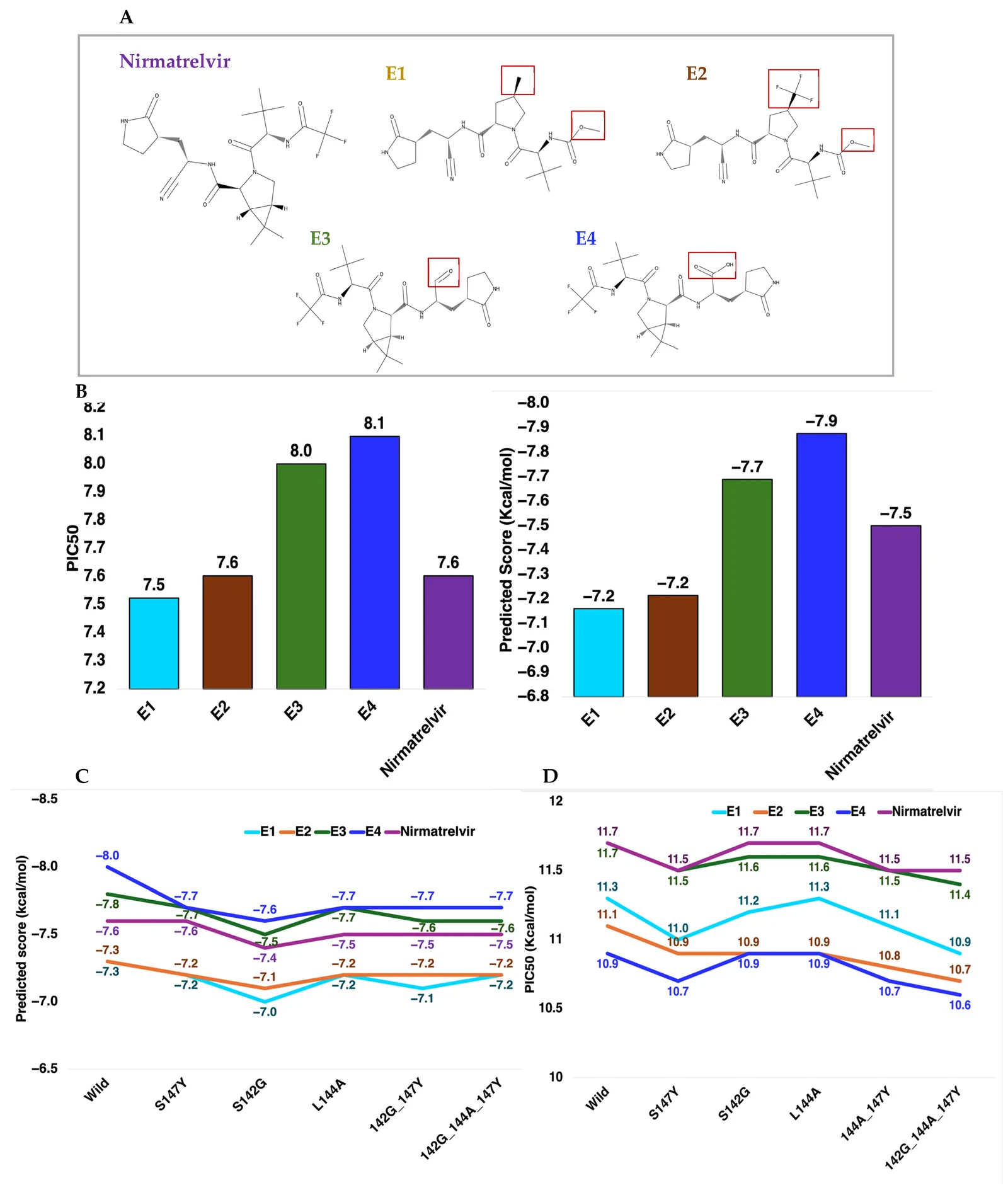
Evaluation of the ensemble model using experimentally validated inhibitors. (**A**) 2D structures of Nirmatrelvir and E1–E4, with their modification sites highlighted by red boxes. (**B**) Experimentally determined pIC_50_ values for the selected inhibitors and ensemble-model-predicted scores (kcal/mol) for E1–E4 and Nirmatrelvir. (**C**) Ensemble-model-predicted scores (kcal/mol) of the selected compounds against wild-type and mutant MERS-CoV Mpro variants, including S142G, L144A, S147Y, S142G+S147Y, and S142G+L144A+S147Y. (**D**) Boltz-2-predicted pIC_50_ values for the same compounds across the corresponding wild-type and mutant MERS-CoV Mpro variants.

To evaluate the predictive capability of the ensemble machine learning model, four Nirmatrelvir derivatives (E1–E4) were assessed and compared with the parent inhibitor, Nirmatrelvir. The chemical structures of the designed analogs are shown in Figure 12A, where the highlighted regions indicate the structural modifications introduced relative to the parent scaffold. Two of these compounds, E1 and E2, were generated by modifying two positions in Nirmatrelvir [40]. The IC_50_ values of E1 and E2 against SARS-CoV-2 Mpro are 0.030 μM and <0.025 μM, respectively. E3 and E4 were generated by replacing the nitrile (-CN) of Nirmatrelvir with carboxylic acid (-COOH) and aldehyde (-CHO) groups, respectively, and they have IC_50_ values of 0.010 μM and 0.008 μM, respectively [41].

The experimental antiviral activities, expressed as pIC50, together with the ensemble model-predicted scores are presented in Figure 12B. The predicted scores closely paralleled the experimental inhibitory pIC50 values, with compounds exhibiting higher pIC50 values also showing more favorable (more negative) predicted scores. The experimental pIC50 values against SARS-CoV-2 for E1, E2, E3, E4 and Nirmatrelvir were 7.5, 7.6, 8.0, 8.1 and 7.6, respectively, while the ensemble model predicted scores were −7.2, −7.2, −7.7, −7.9 and −7.5 kcal/mol, respectively. Although pIC50 primarily reflects inhibitory potency rather than direct binding affinity, the observed correlation indicates that the ensemble model accurately captured the relative binding strengths of this series of inhibitors. Notably, E4 consistently ranked as the strongest binder and most potent inhibitor, while E1 displayed the weakest predicted score and lowest experimental activity.

To assess the impact of resistance-associated mutations, the compounds were evaluated against the wild-type MERS-CoV Mpro and five variants (S147Y, S142G, L144A, S142G/S147Y, and S142G/L144A/S147Y) with both the ensemble model and Boltz-2. Boltz-2 binding affinity prediction was used as an additional computational approach to evaluate the consistency of mutation-dependent trends predicted by the ensemble model. Agreement between the two approaches was interpreted as computational concordance and not as independent experimental validation. As shown in Figure 12C,D, only minor changes in predicted score were observed across all variants with both methods. The ensemble model predicted that all variants displayed a similar pattern of modest reductions in binding scores compared with the wild-type protein for each compound (Figure 12C). Among the compounds, E4 consistently exhibited the most favorable (most negative) binding score (approximately −8.0 to −7.7 kcal/mol) across all variants, followed by E3 (−7.8 to −7.6 kcal/mol) and Nirmatrelvir (−7.6 to −7.5 kcal/mol). E2 showed intermediate binding affinities (−7.3 to −7.1 kcal/mol), while E1 generally displayed the weakest binding (−7.2 to −7.0 kcal/mol). Consistent with the ensemble model, Boltz-2 predicted similar binding affinity trends across the wild-type and mutant proteins, with only slight reductions observed for all variants (Figure 12D). Overall, the ranking of ligand binding scores was largely preserved across all protein variants, following the order E4 > E3 > Nirmatrelvir > E2~E1, demonstrating that the investigated mutations had minimal influence on the relative binding preferences. Boltz-2 also produced highly consistent rankings across all MERS-CoV Mpro variants; however, the overall ranking (Nirmatrelvir ≈ E3 > E1 > E2 > E4) differed from both the ensemble model and the experimental pIC50 values. These discrepancies may reflect known limitations of Boltz-2, including its reduced ability to accurately model large or stereochemically complex ligands, capture ligand-induced conformational changes, and predict binding in allosteric or highly flexible protein regions [42]. Overall, the ensemble model demonstrated superior predictive performance by accurately reproducing the experimental ranking of inhibitor potency while maintaining consistent binding score predictions across all MERS-CoV Mpro variants. These results highlight its robustness and reliability for identifying potent and mutation-tolerant Mpro inhibitors.

## 3. Methods

### 3.1. Molecular Dataset and Feature Extraction

Using RDKit (version 2025.03.3) [26], an open-source cheminformatics toolkit implemented in Python (version 3.12.9) [43], a virtual library of 15,889 Nirmatrelvir derivatives was generated through substitutions at six positions of Nirmatrelvir (Figure 3A). The 3d structures of the ligands were generated from their Simplified Molecular Input Line Entry System (SMILES) representations using OpenBabel (version 3.1.0) [44]. Subsequently, all compounds were docked against the MERS-CoV main protease (Mpro; PDB ID: 7VTC) using AutoDock Vina 1.2.0 [27]. As the analogue library included both nitrile-containing and non-nitrile compounds (Figure 3A), a standardized noncovalent Vina protocol was applied to provide a consistent scoring framework across the complete dataset. To validate the docking protocol, Nirmatrelvir was re-docked into MERS-CoV Mpro (PDB ID: 7VTC) using the same docking parameters. Superimposition of the re-docked and crystallographic poses showed consistent binding orientations within the active site (Supplementary Materials Figure S1). This approach does not explicitly model reversible covalent bond formation between the Nirmatrelvir nitrile warhead and catalytic cysteine and should therefore be considered a limitation of the docking-derived surrogate scores. All molecules were then represented using canonical SMILES notation, and molecular features were generated using RDKit (v2024.09.5) [28]. For each molecule, a comprehensive set of 217 descriptors including constitutional, topological, electronic, and fragment-based properties was computed. To reduce redundancy and improve model efficiency, pairwise correlation analysis was performed, and highly correlated descriptors were removed.

To incorporate protein–ligand interactions, the active-site residues of MERS-CoV Mpro were encoded using residue-level physicochemical descriptors such as charge, polarity, hydrophobicity, and aromaticity. This representation enables the model to capture variations in the binding environment and their influence on ligand docking score. In addition, molecular graph representations were constructed from SMILES using smiles2graph module by OGB-LSC [29], where atoms and bonds are treated as nodes and edges with associated feature vectors describing atomic type, hybridization, bond type, and stereochemistry. These complementary representations allow machine learning models to learn both global molecular properties and local structural interactions.

### 3.2. Machine Learning Model Training and Evaluation

Binding affinities of protein–ligand complexes are influenced by both the ligand properties, such as aromaticity, functional groups, electrostatics, etc., and the protein–ligand interactions, such as residue-level polarity, hydrophobicity, hydrogen-bonding capacity, and conformational flexibility of the protein–ligand system. Favorable hydrophobic and hydrogen-bond interactions generally improve binding, whereas steric hindrance and electrostatic repulsion can reduce affinity of the protein–ligand complex [45]. Molecular descriptors were systematically extracted using RDKit (v2024.09.5) [28], providing a comprehensive representation of ligand properties, including atom and bond counts, partial charges, ring features, topological indices, and polar surface area. To capture protein-specific information, physicochemical properties (hydrophobicity, charge, aromaticity, and polarity) of the target protein’s active-site residues were encoded as numerical features, enabling the models to account for variations in the binding environment. These four properties were selected to provide a compact and interpretable representation of major mutation-induced changes in the local physicochemical environment while limiting feature dimensionality. Additional properties such as residue volume, hydrogen-bonding capacity, solvent accessibility, side-chain flexibility, and evolutionary conservation may further improve mutation representation and represent potential extensions of the framework. Moreover, these ligand and protein features were subsequently integrated to train machine learning models capable of learning complex, non-linear relationships between active-site characteristics and ligand docking scores. Tree-based methods such as CatBoost [37], LightGBM [36], and Random Forest [35] effectively capture feature interactions and handle heterogeneous data, while neural networks like multilayer perceptrons (MLP) [39] were used to capture higher-order non-linear relationships. Additionally, GNNs [46] were evaluated for their ability to learn molecular representations directly from graph-structured data, enabling the capture of complex structural relationships between atoms and chemical bonds.

A multi-strategy machine learning framework was implemented to predict docking score by progressively incorporating ligand and protein information. Using three different approaches, ML models were trained to predict the binding score of ligands to protein; these approaches include: ligand-only framework, integrated ligand–protein framework, and mutation-aware framework.

Within the ligand-only machine learning framework, binding score prediction was formulated as a supervised learning task in which RDKit-derived molecular descriptors served as input features and docking scores as target labels for training RF, XGBoost, LightGBM, CatBoost, MLP, long short-term memory (LSTM), and GNN models. The dataset was split into training, validation, and test sets in an 82:8:10 ratio. Model performance was evaluated using the root mean square error (RMSE), mean absolute error (MAE), and coefficient of determination (R^2^).

Based on their initial predictive performance, the tree-based models Random Forest (RF), XGBoost, LightGBM, and CatBoost, along with the neural network models MLP and GNN, were selected for further evaluation in the integrated ligand–protein modeling framework. In this framework, RDKit-derived ligand descriptors were combined with encoded active-site features derived from 27 key protein residues. Each residue was represented using physicochemical properties, including hydrophobicity, charge, aromaticity, and polarity, enabling the models to capture interaction-dependent relationships between ligand structure and the protein binding environment.

Finally, a mutation-aware modeling strategy was employed to explicitly account for active-site variability. In this approach, protein sequences were encoded relative to a reference (wild-type) sequence using a mutation-delta representation, in which each residue was described by a set of physicochemical properties, including hydrophobicity, charge, aromaticity, and polarity. For each residue position, the feature values of the wild-type amino acid were subtracted from those of the corresponding mutant amino acid, producing a delta feature vector that quantitatively represents the physicochemical changes introduced by the mutation. Residue positions without mutations were assigned zero-valued delta vectors, ensuring that only mutation-induced changes contributed to the protein representation. This encoding enabled the model to focus on the effects of amino acid substitutions while preserving the positional information of the active-site residues.

A two-stage learning framework was then employed, in which a ligand baseline model first predicted the average binding score of each ligand independent of the protein sequence. A mutation model subsequently learned the residual change in docking score associated with the mutation-delta features. The final docking-score prediction was obtained by combining the baseline ligand prediction with the mutation-induced correction, allowing the framework to capture both ligand-specific and mutation-dependent effects on protein–ligand binding. To ensure unbiased evaluation and prevent data leakage, ligand-grouped data splitting was applied, where all variants of a given ligand were confined to either training or test sets.

In the original approach, Stage B used only the delta-encoded mutation features, resulting in an additive approach:

(1) $$y=f\left(X_{L}\right)+g(∆P_{M})$$

where y is the predicted score for ligand L against protein variant M; X_L_ represents the molecular descriptor vector of ligand L; ΔP_M_ represents the delta-encoded physicochemical changes of variant M relative to the wild-type active-site sequence; f(X_L_) represents the Stage A ligand-level baseline prediction; and g(ΔP_M_) represents the Stage B mutation-associated residual correction.

To evaluate whether explicit ligand–mutation interactions improve predictive performance, an additional interaction-aware formulation was implemented while retaining Stage A unchanged:

(2) $$y=f\left(X_{L}\right)+h(X_{L},∆P_{M})$$

where h(X_L_,ΔP_M_) represents the interaction-aware Stage B residual model, which jointly incorporates the ligand descriptors and mutation-delta features. Unlike the original g(ΔP_M_) term, this formulation allows the predicted mutation-associated residual to vary according to ligand identity.

### 3.3. Evaluation Using Experimental IC_50_ Data and Boltz-2 Predictions

The ensemble model was evaluated using Nirmatrelvir and four derivatives with experimentally reported IC_50_ values against SARS-CoV-2 Mpro. Experimental IC_50_ values, reported in μM, were converted to pIC_50_ using Equation (3).

(3) $${PIC}_{50}=-log({IC}_{50}\times 10^{-6}M)$$

Higher pIC_50_ values indicate greater inhibitory potency. The experimental pIC_50_ values were compared with the ensemble-predicted scores, for which more negative values indicate stronger predicted binding. It should be noted that pIC50 and binding affinity are not directly equivalent; therefore, a higher pIC50 value does not always indicate stronger binding.

The ensemble model was further evaluated against Boltz-2, which has demonstrated performance comparable to physics-based free-energy perturbation methods and superior to several existing machine learning approaches [47]. Boltz-2 predictions were generated for the same compounds against wild-type MERS-CoV Mpro and five mutant variants. Boltz-2 predicts binding affinity, y, as log10(IC_50_), which was converted to pIC_50_ in kcal/mol using Equation (4), where higher pIC_50_ values indicate stronger predicted binding.

(4) $$pIC50=\left(6-y\right)\times 1.364$$

## 4. Web Interface for Binding Score Prediction

A user-friendly web interface was developed to enable rapid prediction of ligand binding score using the optimized machine learning ensemble model (Supplementary Materials Figure S2). The platform accepts a canonical SMILES string for the ligand and a protein PDB file as inputs. Upon PDB upload, the 27 predefined active-site positions surrounding the Mpro binding pocket are automatically identified, and their corresponding residue information is extracted from the structure. These protein features, together with the calculated ligand descriptors, are then provided to the ensemble model to generate the predicted score. To accommodate PDB structures with different residue-numbering schemes and facilitate mutation analysis, the interface displays the residue number and corresponding one-letter amino-acid code for each of the 27 active-site positions. Both fields can be edited by the user. Changing a residue number maps that active-site position to the specified residue in the uploaded PDB structure, whereas changing the amino-acid code defines a point mutation at that position. This feature enables users to evaluate the predicted effect of active-site mutations without preparing and uploading a separate mutant PDB structure. The platform supports both single-molecule and batch predictions. For individual predictions, users upload a PDB structure and provide a ligand SMILES string, whereas multiple compounds can be evaluated through CSV file upload. Prediction results can subsequently be downloaded in CSV format for further analysis. Overall, the web interface integrates automated active-site extraction, user-defined residue mapping and mutation analysis, and the optimized ensemble model into an accessible workflow for evaluating ligand binding and mutation-dependent changes in predicted score across Mpro–ligand systems, thereby supporting virtual screening and structure-guided drug design.

## 5. Conclusions

In this study, we developed a mutation-aware machine learning framework for predicting the binding affinities of Nirmatrelvir analogues against wild-type and mutant coronavirus main proteases. By progressively integrating ligand descriptors, active-site features, and mutation-specific information, the framework captured both the intrinsic molecular properties of the ligands and the mutation-dependent effects governing their interactions with the target protein. A key innovation was the mutation-delta strategy, which decomposed the docking score into ligand-baseline and mutation-induced contributions, thereby improving predictive accuracy and interpretability. Among the individual models, tree-based algorithms exhibited the strongest predictive performance, with CatBoost achieving a MAE of 0.23 kcal/mol and an R^2^ of 0.87. The weighted ensemble model further improved predictive accuracy, yielding a MAE of 0.19 kcal/mol and an R^2^ of 0.90. Evaluation using an additional dataset of 1000 newly generated Nirmatrelvir analogues further demonstrated the generalizability of the framework. The ensemble model achieved the best predictive performance with the lowest MAE (0.29 kcal/mol) and the highest R^2^ (0.78), followed closely by CatBoost (MAE = 0.29 kcal/mol, R^2^ = 0.77), outperforming all other evaluated machine learning models. The robustness and generalizability of the mutation-aware ensemble framework were further demonstrated through random, ligand-group, and scaffold-group cross-validation, where it achieved an R^2^ of 0.94 and MAE of 0.12 kcal/mol under random and ligand-group splits, while maintaining comparable performance under the scaffold-group split (R^2^ = 0.92, MAE = 0.13 kcal/mol). Furthermore, the negative R^2^ values obtained from Y-randomization (−0.93 to −0.99), together with substantially higher prediction errors, confirmed that the model learned meaningful mutation-dependent protein–ligand relationships rather than random correlations.

SHAP analysis identified 20 key molecular descriptors contributing to the predictions and provided insight into the molecular features governing ligand binding. Importantly, comparison with selected experimentally characterized Nirmatrelvir derivatives showed qualitative consistency between the predicted values and reported inhibitor-potency trends, with experimental pIC_50_ values ranging from 7.5 to 8.1 and predicted scores ranging from −7.2 to −7.9 kcal/mol. Across the wild-type and five mutant MERS-CoV Mpro variants, the model consistently predicted only modest mutation-dependent changes in binding score while preserving the relative ranking of the inhibitors. The ensemble model and Boltz-2 showed broadly similar mutation-dependent trends across the MERS-CoV Mpro variants, although the absolute ranking of individual compounds differed between the two computational approaches. Collectively, these results demonstrate that the developed framework is scalable, interpretable, and sensitive to active-site mutations, supporting its application in the prioritization and rational optimization of antiviral inhibitors against emerging drug-resistant coronavirus variants.

## Figures and Tables

**Table 1:** Descriptions of important ligand features identified from SHapley Additive exPlanations (SHAP) analysis.

Feature Name	Description
HeavyAtomMolWt	Molecular weight contributed by heavy (non-hydrogen) atoms, reflecting overall molecular size.
Chi0	Zeroth-order molecular connectivity index, representing molecular branching and topology.
HeavyAtomCount	Total number of heavy atoms in the molecule, indicating molecular complexity.
MolWt	Total molecular weight of the compound.
NumValenceElectrons	Total number of valence electrons, related to bonding capacity and reactivity.
ExactMolWt	Exact molecular weight based on isotopic masses.
Ipc	Information content index, measuring molecular complexity and symmetry.
LabuteASA	Approximate surface area (Labute method), related to molecular size and exposure.
BertzCT	Bertz complexity index, quantifying structural complexity.
Chi1	First-order molecular connectivity index, capturing connectivity patterns between atoms.
BalabanJ	Balaban’s J index, a topological descriptor reflecting molecular shape and branching.
RingCount	Number of rings in the molecule, indicating cyclic structure presence.
HallKierAlpha	Hall–Kier alpha index, reflecting atomic contributions to molecular connectivity and shape.
NumAmideBonds	Number of amide bonds (-C(=O)N-), important for hydrogen bonding and stability.
prot_pos4_hydro	Hydrophobic property change at protein position 4, indicating mutation-induced hydrophobic shifts.
Chi0n	Normalized zeroth-order connectivity index, adjusted for molecular size.
prot_pos4_polar	Polarity-related change at protein position 4 due to mutation.
MolMR	Molecular refractivity, related to volume and polarizability of the molecule.
Kappa1	First kappa shape index, describing molecular shape and flexibility.
AvgIpc	Average information content per atom, representing normalized molecular complexity.

**Table 2:** Descriptions of important protein features identified from SHapley Additive exPlanations (SHAP) analysis.

Feature Name	Description
prot_pos4_hydro	Change in hydrophobicity at protein position 4 due to mutation, influencing ligand binding interactions.
prot_pos4_polar	Change in polarity at protein position 4, affecting hydrogen bonding and electrostatic interactions.
prot_pos6_hydro	Hydrophobicity variation at protein position 6 caused by mutation, impacting local binding environment.
prot_pos9_arom	Change in aromatic character at protein position 9, influencing π–π stacking and aromatic interactions.
prot_pos9_hydro	Hydrophobicity change at protein position 9, affecting ligand affinity and pocket environment.

## Data Availability

All machine learning codes, datasets, and scripts used in this study are deposited and shared in the GitHub repository maintained by our research group: https://github.com/Dr-Islam-Lab-Group/Binding-affinity-prediction (accessed on 11 February 2026). The repository includes model training pipelines, prediction tools, processed datasets, and instructions to reproduce the results presented in this work. Web interface to predict binding score: https://sites.google.com/view/shahidulislamlab/utilities/binding-affinity-predictor (accessed on 11 February 2026).

## Supplementary Materials

The following Supporting Information can be downloaded at: https://www.mdpi.com/article/10.3390/molecules31172949/s1, Supporting Information includes Table S1. Performance comparison of ligand-only models on the test dataset using mean absolute error (MAE) and coefficient of determination (R^2^). Results represent the best performance achieved across five independent training and testing runs; Table S2. Comparison of the mean absolute error (MAE; kcal/mol) of ligand-only models inpredicting the docking-scores of 1000 previously held out nirmatrelvir analogues; Table S3. Comparison of the regression coefficient (R^2^), and mean absolute error (MAE; kcal/mol)of the integrated ligand-protein modeling framework on the test dataset; Table S4. Comparison of the mean absolute error (MAE; kcal/mol) of the integrated ligand–protein models in predicting the docking-derived binding affinities of 1000 previously held-out nirmatrelvir analogues; Table S5: List of all the RDkit descriptors extracted from each molecular SMILES; Table S6: Performance metrics of five replica runs for each optimized ML model for mutation aware approach; Table S7: Performance of weighted ensemble models combining the best trained ML models and their corresponding prediction accuracy metrics; Table S8. Comparison of the regression coefficient (R^2^), and mean absolute error (MAE; kcal/mol) of the ligand–mutation interaction-aware modeling on the test dataset; Table S9. Summary of structural modifications introduced into 1000 additional Nirmatrelvir analogues; Table S10. Individual fold performance of the CatBoost and ensemble (CatBoost + XGBoost) models under repeated random 5-fold cross-validation, ligand-based GroupKFold, and Murcko scaffold-based GroupKFold. Test-set performance is reported as the coefficient of determination (R^2^), root mean square error (RMSE) and mean absolute error (MAE). Repeated random 5-fold cross-validation comprised 25 train–test splits, whereas ligand-based and scaffold-based GroupKFold each consisted of 5 independent folds; Table S11. Performance of the CatBoost and ensemble (CatBoost + XGBoost) models following Y-randomization across repeated random 5-fold, ligand-based GroupKFold, and Murcko scaffold-based GroupKFold. Docking score labels were randomly shuffled 20 times for each validation strategy, and model performance is reported as R^2^, RMSE, and MAE; Figure S1. Docking validation and binding mode comparison of Nirmatrelvir analogues with MERS-CoV main protease. Superimposition of the crystallographic ligand (blue) and the re-docked pose (yellow) within the active site of the MERS-CoV main protease. The RMSD calculated over 35 corresponding atom pairs was 5.6 Å, indicating that the re-docked pose occupies the same binding region but shows conformational deviation from the crystallographic binding orientation; Figure S2: Web interface to predict binding score values (https://sites.google.com/view/shahidulislamlab/utilities/binding-affinity-predictor).
