# Supplementary material for: Mutation-Aware Machine Learning Framework for Predicting Binding Affinity of Nirmatrelvir Analogs Targeting Coronavirus Main Proteases
Source: Molecules. 2026 Aug 22;31(17):2949. doi: 10.3390/molecules31172949 (PMC13567233; doi:10.3390/molecules31172949)
Supplement: Supplementary file 1 [file molecules-31-02949-s001.zip › Supporting information/Supporting_Information_updated.pdf]

Supporting Information

Mutation-Aware Machine Learning Framework for Predicting Binding-Affinity of Nirmatrelvir Analogs Targeting Corona Virus Main Proteases

Md Saidur Rahman[1], Md Mehedi Hasan[1], Shahidul M. Islam[1*]

[1]Department of Chemistry, Delaware State University, Dover, DE 19901, USA

*Corresponding:

E-mail: sislam@desu.edu, Tel. 302-857-7181

Table S1. Performance comparison of ligand-only models on the test dataset using mean absolute error (MAE) and coefficient of determination (R^2). Results represent the best performance achieved across five independent training and testing runs.

**Model name**	**Evaluation matrix**	
	**MAE**	**R^2**
Graph Neural Network	0.32	0.76
LSTM	0.35	0.82
Multilayer perceptron (MLP)	0.24	0.85
CatBoost	0.23	0.85
Random forest (RF)	0.23	0.86
LGBM	0.22	0.86
[a]Ensemble_LGBM_CatBoost_LSTM	0.22	0.86
[b]Ensemble_MLP_RF_LGBM	0.21	0.87
[c]Ensemble_RF_CatBoost_LGBM	0.21	0.88

[a]Model weight ratio 0.8:0.2:0.0; [b]Model weight ratio 0.0:0.1:0.9; [c]Model weight ratio 0.1:0.1:0.8.

Table S2. Comparison of the mean absolute error (MAE; kcal/mol) of ligand-only models in predicting the docking-scores of 1,000 previously held out nirmatrelvir analogues.

**Model name**	**Evaluation matrix**	
	**MAE**	**R^2**
Graph Neural Network	0.34	0.68
Multilayer perceptron	0.29	0.76
Random forest	0.30	0.76
LGBM	0.33	0.69
Catboost	0.48	0.38
LSTM	0.84	0.60
[a]Ensemble_LGBM_CatBoost_LSTM	0.38	0.61
[b]Ensemble_MLP_RF_LGBM	0.32	0.71
[c]Ensemble_RF_CatBoost_LGBM	0.31	0.75

[a]Model weight ratio 0.8:0.2:0.0; [b]Model weight ratio 0.0:0.1:0.9; [c]Model weight ratio 0.1:0.1:0.8.

Table S3. Comparison of the regression coefficient (R^2), and mean absolute error (MAE; kcal/mol) of the integrated ligand-protein modeling framework on the test dataset.

Model Name	Evaluation matrix	
	MAE	R2
Graph Neural Network	0.25	0.83
Multilayer perceptron	0.25	0.84
XGB	0.23	0.85
Random forest (RF)	0.22	0.86
LGBM	0.23	0.85
Catboost (CB)	0.22	0.86
[a]Ensemble_RF_LGBM_CatBoost	0.22	0.86

[a]Model weight ratio 0.3:0.0:0.7.

Table S4. Comparison of the mean absolute error (MAE; kcal/mol) of the integrated ligand–protein models in predicting the docking-derived binding affinities of 1,000 previously held-out nirmatrelvir analogues.

Model Name	Evaluation matrix	
	MAE	R2
Graph Neural Network	0.31	0.74
Multilayer perceptron	0.30	0.75
XGB	0.30	0.74
Random forest	0.30	0.75
LGBM	0.29	0.76
Catboost	0.28	0.77
[a]Ensemble_RF_LGBM_CatBoost	0.28	0.77

[a]Model weight ratio 0.3:0.0:0.7.

Table S5: List of all the RDkit descriptors extracted from each molecular SMILES.

Descriptors Name		Selected Descriptors for training models
MaxAbsEStateIndex	NumAliphaticCarbocycles	SPS
MaxEStateIndex	NumAliphaticHeterocycles	MolWt
MinAbsEStateIndex	NumAliphaticRings	HeavyAtomMolWt
MinEStateIndex	NumAmideBonds	ExactMolWt
qed	NumAromaticCarbocycles	NumValenceElectrons
SPS	NumAromaticHeterocycles	BCUT2D_MWHI
MolWt	NumAromaticRings	BertzCT
HeavyAtomMolWt	NumAtomStereoCenters	Chi0
ExactMolWt	NumBridgeheadAtoms	Chi0n
NumValenceElectrons	NumHAcceptors	Chi0v
NumRadicalElectrons	NumHDonors	Chi1
MaxPartialCharge	NumHeteroatoms	Chi1n
MinPartialCharge	NumHeterocycles	Chi1v
MaxAbsPartialCharge	NumRotatableBonds	Chi2n
MinAbsPartialCharge	NumSaturatedCarbocycles	Chi2v
FpDensityMorgan1	NumSaturatedHeterocycles	Chi3v
FpDensityMorgan2	NumSaturatedRings	Ipc
FpDensityMorgan3	NumSpiroAtoms	Kappa1
BCUT2D_MWHI	NumUnspecifiedAtomStereoCenters	Kappa2
BCUT2D_MWLOW	Phi	LabuteASA
BCUT2D_CHGHI	RingCount	PEOE_VSA1
BCUT2D_CHGLO	MolLogP	PEOE_VSA10
BCUT2D_LOGPHI	MolMR	PEOE_VSA12
BCUT2D_LOGPLOW	fr_Al_COO	PEOE_VSA14
BCUT2D_MRHI	fr_Al_OH	PEOE_VSA2
BCUT2D_MRLOW	fr_Al_OH_noTert	PEOE_VSA3
AvgIpc	fr_ArN	PEOE_VSA4
BalabanJ	fr_Ar_COO	PEOE_VSA5
BertzCT	fr_Ar_N	PEOE_VSA6
Chi0	fr_Ar_NH	PEOE_VSA7
Chi0n	fr_Ar_OH	PEOE_VSA8

Chi0v	fr_COO	PEOE_VSA9
Chi1	fr_COO2	SMR_VSA1
Chi1n	fr_C_O	SMR_VSA10
Chi1v	fr_C_O_noCOO	SMR_VSA2
Chi2n	fr_C_S	SMR_VSA3
Chi2v	fr_HOCCN	SMR_VSA4
Chi3n	fr_Imine	SMR_VSA5
Chi3v	fr_NH0	SMR_VSA6
Chi4n	fr_NH1	SMR_VSA7
Chi4v	fr_NH2	SMR_VSA9
HallKierAlpha	fr_N_O	SlogP_VSA1
Ipc	fr_Ndealkylation1	SlogP_VSA10
Kappa1	fr_Ndealkylation2	SlogP_VSA12
Kappa2	fr_Nhpyrrole	SlogP_VSA2
Kappa3	fr_SH	SlogP_VSA3
LabuteASA	fr_aldehyde	SlogP_VSA4
PEOE_VSA1	fr_alkyl_carbamate	SlogP_VSA5
PEOE_VSA10	fr_alkyl_halide	SlogP_VSA6
PEOE_VSA11	fr_allylic_oxid	TPSA
PEOE_VSA12	fr_amide	EState_VSA1
PEOE_VSA13	fr_amidine	EState_VSA10
PEOE_VSA14	fr_aniline	EState_VSA2
PEOE_VSA2	fr_aryl_methyl	EState_VSA3
PEOE_VSA3	fr_azide	EState_VSA4
PEOE_VSA4	fr_azo	EState_VSA5
PEOE_VSA5	fr_barbitur	EState_VSA6
PEOE_VSA6	fr_benzene	EState_VSA7
PEOE_VSA7	fr_benzodiazepine	EState_VSA8
PEOE_VSA8	fr_bicyclic	EState_VSA9
PEOE_VSA9	fr_diazo	VSA_EState1
SMR_VSA1	fr_dihydropyridine	VSA_EState2
SMR_VSA10	fr_epoxide	VSA_EState3
SMR_VSA2	fr_ester	VSA_EState4

SMR_VSA3	fr_ether	VSA_EState5
SMR_VSA4	fr_furan	VSA_EState6
SMR_VSA5	fr_guanido	VSA_EState7
SMR_VSA6	fr_halogen	VSA_EState8
SMR_VSA7	fr_hdrzine	HeavyAtomCount
SMR_VSA8	fr_hdrzone	NOCount
SMR_VSA9	fr_imidazole	NumHeteroatoms
SlogP_VSA1	fr_imide	NumRotatableBonds
SlogP_VSA10	fr_isocyan	MolMR
SlogP_VSA11	fr_isothiocyan	
SlogP_VSA12	fr_ketone	
SlogP_VSA2	fr_ketone_Topliss	
SlogP_VSA3	fr_lactam	
SlogP_VSA4	fr_lactone	
SlogP_VSA5	fr_methoxy	
SlogP_VSA6	fr_morpholine	
SlogP_VSA7	fr_nitrile	
SlogP_VSA8	fr_nitro	
SlogP_VSA9	fr_nitro_arom	
TPSA	fr_nitro_arom_nonortho	
EState_VSA1	fr_nitroso	
EState_VSA10	fr_oxazole	
EState_VSA11	fr_oxime	
EState_VSA2	fr_para_hydroxylation	
EState_VSA3	fr_phenol	
EState_VSA4	fr_phenol_noOrthoHbond	
EState_VSA5	fr_phos_acid	
EState_VSA6	fr_phos_ester	
EState_VSA7	fr_piperdine	
EState_VSA8	fr_piperzine	
EState_VSA9	fr_priamide	
VSA_EState1	fr_prisulfonamd	
VSA_EState10	fr_pyridine	

VSA_EState2	fr_quatN	
VSA_EState3	fr_sulfide	
VSA_EState4	fr_sulfonamd	
VSA_EState5	fr_sulfone	
VSA_EState6	fr_term_acetylene	
VSA_EState7	fr_tetrazole	
VSA_EState8	fr_thiazole	
VSA_EState9	fr_thiocyan	
FractionCSP3	fr_thiophene	
HeavyAtomCount	fr_unbrch_alkane	
NHOHCount	fr_urea	
NOCount		

Table S6: Performance metrics of five replica runs for each optimized ML model for mutation aware approach.

		**Evaluation Metrics**		
**Model Name**	**Kfold (K=5)**	**MAE**	**R2**	**RMSE**
**XGBoost Regressor**	fold1	0.27	0.78	0.36
	fold2	**0.26**	**0.82**	**0.34**
	fold3	0.26	0.83	0.35
	fold4	0.27	0.82	0.35
	fold5	0.26	0.81	0.34
	Avg.	0.27	0.81	0.35
**Random Forest Regressor**	fold1	0.27	0.78	0.35
	fold2	0.26	0.82	0.34
	fold3	0.27	0.82	0.36
	fold4	**0.26**	**0.82**	**0.34**
	fold5	0.27	0.80	0.35
	Avg.	0.27	0.81	0.35
**LGBM Regressor**	fold1	0.27	0.78	0.35
	fold2	0.27	0.81	0.34
	fold3	**0.27**	**0.83**	**0.35**
	fold4	0.28	0.81	0.36
	fold5	0.27	0.80	0.35
	Avg.	0.27	0.81	0.35

**CatBoost Regressor**	fold1	0.23	0.83	0.31
	fold2	0.24	0.84	0.31
	fold3	**0.23**	**0.87**	**0.31**
	fold4	0.23	0.86	0.31
	fold5	0.24	0.84	0.31
	Avg.	0.24	0.85	0.31
**Graph Neural Network (GNN)**	fold1	0.34	0.66	0.44
	fold2	0.31	0.76	0.39
	fold3	0.32	0.78	0.40
	fold4	**0.30**	**0.78**	**0.38**
	fold5	0.30	0.76	0.38
	Avg.	0.31	0.75	0.40
**Multilayer Perceptron**	fold1	0.31	0.72	0.40
	fold2	0.28	0.79	0.36
	fold3	**0.27**	**0.84**	**0.35**
	fold4	0.28	0.80	0.36
	fold5	0.28	0.79	0.36
	Avg.	0.28	0.79	0.36

*Highlighted results represent the best performance of each model across the five runs.

Table S7: Performance of weighted ensemble models combining the best trained ML models and their corresponding prediction accuracy metrics.

CatBoost Weight	XGB Weight	LGBMWeight	RMSE	MAE	R^2
0	0	1	0.3129	0.2387	0.8568
0	0.1	0.9	0.3066	0.2334	0.8625
0	0.2	0.8	0.3009	0.2286	0.8676
0	0.3	0.7	0.2956	0.2239	0.8722
0	0.4	0.6	0.2908	0.2194	0.8763
0	0.5	0.5	0.2865	0.2153	0.8799
0	0.6	0.4	0.2828	0.2117	0.8831
0	0.7	0.3	0.2796	0.2086	0.8857
0	0.8	0.2	0.2770	0.2061	0.8877
0	0.9	0.1	0.2751	0.2042	0.8893
0	1	0	0.2737	0.2030	0.8904
0.1	0	0.9	0.3043	0.2316	0.8646

0.1	0.1	0.8	0.2985	0.2266	0.8697
0.1	0.2	0.7	0.2932	0.2219	0.8743
0.1	0.3	0.6	0.2883	0.2174	0.8784
0.1	0.4	0.5	0.2840	0.2132	0.8820
0.1	0.5	0.4	0.2803	0.2097	0.8851
0.1	0.6	0.3	0.2771	0.2065	0.8877
0.1	0.7	0.2	0.2745	0.2040	0.8898
0.1	0.8	0.1	0.2725	0.2021	0.8914
0.1	0.9	0	0.2712	0.2009	0.8924
0.2	0	0.8	0.2963	0.2247	0.8716
0.2	0.1	0.7	0.2909	0.2199	0.8762
0.2	0.2	0.6	0.2861	0.2155	0.8803
0.2	0.3	0.5	0.2818	0.2114	0.8839
0.2	0.4	0.4	0.2780	0.2078	0.8870
0.2	0.5	0.3	0.2748	0.2047	0.8895
0.2	0.6	0.2	0.2722	0.2022	0.8916
0.2	0.7	0.1	0.2702	0.2002	0.8932
0.2	0.8	0	0.2689	0.1990	0.8943
0.3	0	0.7	0.2890	0.2182	0.8779
0.3	0.1	0.6	0.2841	0.2137	0.8820
0.3	0.2	0.5	0.2797	0.2097	0.8855
0.3	0.3	0.4	0.2759	0.2061	0.8886
0.3	0.4	0.3	0.2727	0.2030	0.8912
0.3	0.5	0.2	0.2701	0.2005	0.8933
0.3	0.6	0.1	0.2682	0.1985	0.8948
0.3	0.7	0	0.2668	0.1972	0.8959
0.4	0	0.6	0.2823	0.2122	0.8834
0.4	0.1	0.5	0.2779	0.2082	0.8870
0.4	0.2	0.4	0.2741	0.2046	0.8901
0.4	0.3	0.3	0.2709	0.2015	0.8926
0.4	0.4	0.2	0.2683	0.1990	0.8947
0.4	0.5	0.1	0.2663	0.1969	0.8963
0.4	0.6	0	0.2650	0.1957	0.8973
0.5	0	0.5	0.2764	0.2069	0.8883
0.5	0.1	0.4	0.2726	0.2033	0.8913
0.5	0.2	0.3	0.2693	0.2002	0.8939
0.5	0.3	0.2	0.2667	0.1976	0.8959
0.5	0.4	0.1	0.2648	0.1956	0.8975
0.5	0.5	0	0.2634	0.1944	0.8985

0.6	0	0.4	0.2712	0.2023	0.8924
0.6	0.1	0.3	0.2680	0.1992	0.8949
0.6	0.2	0.2	0.2654	0.1966	0.8970
0.6	0.3	0.1	0.2634	0.1946	0.8985
0.6	0.4	0	0.2621	0.1933	0.8995
0.7	0	0.3	0.2669	0.1984	0.8958
0.7	0.1	0.2	0.2643	0.1958	0.8978
0.7	0.2	0.1	0.2624	0.1938	0.8993
0.7	0.3	0	0.2610	0.1924	0.9003
0.8	0	0.2	0.2635	0.1951	0.8984
0.8	0.2	0	0.2615	0.1931	0.8999
**0.8**	**0.1**	**0.1**	**0.2594**	**0.1914**	**0.9016**
0.9	0	0.1	0.2597	0.1914	0.9014
0.9	0.1	0	0.2610	0.1928	0.9004
1	0	0	0.2602	0.1918	0.9010

Table S8. Comparison of the regression coefficient (R^2), and mean absolute error (MAE; kcal/mol) of the ligand–mutation interaction-aware modeling on the test dataset.

**Model Name**	**Evaluation matrix**	
	**MAE**	**R2**
Multilayer perceptron	0.22	0.84
XGB	0.20	0.86
Random forest (RF)	0.22	0.84
LGBM	0.21	0.86
Catboost (CB)	0.20	0.86
[a]Ensemble_XGB_LGBM_CatBoost	0.20	0.87

[a]Model weight ratio 0.3:0.2:0.5.

Table S9. Summary of structural modifications introduced into 1000 additional Nirmatrelvir analogues.

Nirmatrelvir structural region	Original group	Modified groups
Warhead	–C≡N	$–CSNH_2$
Pyrrolidinone	C=O	C=S
Trifluoroacetamide	$–CF_3$	$–CF_2CN$,$–CF_2NO_2$,–<br>CF_2CONH_2, $–CF_2OCH_3$
Alkyl side chain	$–CH_3$	$–CH_2F$, $–CHF_2$, $–CH_2Cl$
Amide linker	–NH–	–NH–NH–
Terminal side-chain region	Pyrrolidinone	Phenylamide

Table S10. Individual fold performance of the CatBoost and ensemble (CatBoost + XGBoost) models under repeated random 5-fold cross-validation, ligand-based GroupKFold, and Murcko scaffold-based GroupKFold. Test-set performance is reported as the coefficient of determination (R^2), root mean square error (RMSE) and mean absolute error (MAE). Repeated random 5-fold cross-validation comprised 25 train–test splits, whereas ligand-based and scaffold-based GroupKFold each consisted of 5 independent folds.

Strategy	Split	R^2	RMSE	MAE
CatBoost				
**Random_repeatedkfold**	1	0.9295	0.1907	0.1344
	2	0.9321	0.1935	0.1349
	3	0.9329	0.1875	0.1343
	4	0.9353	0.1853	0.1331
	5	0.9292	0.1934	0.1367
	6	0.9309	0.1924	0.1348
	7	0.9326	0.1904	0.1364
	8	0.9288	0.1911	0.1345
	9	0.9326	0.1877	0.1337
	10	0.9309	0.1931	0.1347
	11	0.9395	0.1840	0.1330
	12	0.9332	0.1867	0.1344
	13	0.9247	0.1962	0.1362
	14	0.9292	0.1930	0.1358
	15	0.9337	0.1879	0.1333
	16	0.9282	0.1956	0.1396
	17	0.9367	0.1852	0.1320
	18	0.9316	0.1885	0.1325
	19	0.9328	0.1898	0.1346
	20	0.9314	0.1889	0.1358
	21	0.9278	0.1914	0.1364

	22	0.9331	0.1905	0.1348
	23	0.9315	0.1903	0.1340
	24	0.9332	0.1879	0.1332
	25	0.9311	0.1935	0.1364
**Ligand_groupkfold**	1	0.8874	0.2450	0.1790
	2	0.8918	0.2349	0.1750
	3	0.8887	0.2390	0.1788
	4	0.9034	0.2304	0.1720
	5	0.8946	0.2395	0.1771
**Scaffold_groupkfold_murcko**	1	0.7265	0.2280	0.1749
	2	0.4238	0.3908	0.3159
	3	0.9104	0.2687	0.2102
	4	0.8690	0.2880	0.2183
	5	0.8344	0.3302	0.2588
Ensemble (CatBoost + XGB)				
**Random_repeatedkfold**	1	0.9414	0.1739	0.1225
	2	0.9418	0.1792	0.1241
	3	0.9415	0.1750	0.1237
	4	0.9448	0.1712	0.1225
	5	0.9395	0.1788	0.1270
	6	0.9432	0.1745	0.1227
	7	0.9428	0.1754	0.1251
	8	0.9385	0.1776	0.1244
	9	0.9428	0.1730	0.1234
	10	0.9414	0.1779	0.1243
	11	0.9480	0.1705	0.1235
	12	0.9418	0.1742	0.1238
	13	0.9359	0.1810	0.1253
	14	0.9390	0.1792	0.1247
	15	0.9436	0.1732	0.1225
	16	0.9383	0.1813	0.1281
	17	0.9472	0.1691	0.1204
	18	0.9401	0.1764	0.1230
	19	0.9427	0.1752	0.1240
	20	0.9404	0.1761	0.1243
	21	0.9388	0.1762	0.1255
	22	0.9427	0.1763	0.1241
	23	0.9418	0.1755	0.1226
	24	0.9443	0.1717	0.1217

	25	0.9413	0.1786	0.1260
**Ligand_groupkfold**	1	0.9395	0.1796	0.1275
	2	0.9352	0.1817	0.1266
	3	0.9434	0.1705	0.1214
	4	0.9481	0.1688	0.1216
	5	0.9422	0.1773	0.1229
**Scaffold_groupkfold_murcko**	1	0.8758	0.1536	0.1120
	2	0.9050	0.1587	0.1119
	3	0.9701	0.1551	0.1100
	4	0.9350	0.2029	0.1444
	5	0.9322	0.2113	0.1495

Table S11. Performance of the CatBoost and ensemble (CatBoost + XGBoost) models following Y-randomization across repeated random 5-fold, ligand-based GroupKFold, and Murcko scaffold-based GroupKFold. Docking score labels were randomly shuffled 20 times for each validation strategy, and model performance is reported as R^2, RMSE, and MAE.

strategy	shuffle	CatBoost			Ensemble (CatBoost +XGB)		
		R^2	RMSE	MAE	R^2	RMSE	MAE
**Random_repeatedkfold_y_randomized**	1	-0.1257	0.7723	0.5865	-0.9198	1.0084	0.7722
	2	-0.1220	0.7712	0.5845	-0.9175	1.0081	0.7707
	3	-0.1283	0.7733	0.5868	-0.9159	1.0076	0.7707
	4	-0.1278	0.7730	0.5876	-0.9227	1.0093	0.7712
	5	-0.1327	0.7746	0.5875	-0.9237	1.0094	0.7721
	6	-0.1267	0.7727	0.5872	-0.9430	1.0147	0.7744
	7	-0.1241	0.7718	0.5867	-0.9595	1.0190	0.7792
	8	-0.1280	0.7731	0.5866	-0.9169	1.0077	0.7728
	9	-0.1368	0.7761	0.5889	-0.9438	1.0146	0.7817
	10	-0.1324	0.7745	0.5877	-0.9602	1.0188	0.7760
	11	-0.1223	0.7712	0.5848	-0.9163	1.0076	0.7700
	12	-0.1219	0.7711	0.5861	-0.9459	1.0154	0.7750
	13	-0.1297	0.7737	0.5866	-0.9553	1.0179	0.7779
	14	-0.1345	0.7753	0.5885	-0.9371	1.0131	0.7741
	15	-0.1242	0.7718	0.5845	-0.9400	1.0137	0.7775
	16	-0.1305	0.7739	0.5877	-0.9269	1.0102	0.7718
	17	-0.1269	0.7727	0.5880	-0.9181	1.0080	0.7717
	18	-0.1260	0.7725	0.5877	-0.9250	1.0098	0.7713
	19	-0.1283	0.7732	0.5871	-0.9020	1.0038	0.7704
	20	-0.1272	0.7728	0.5882	-0.9577	1.0183	0.7751

**Ligand_groupkfold_y_randomized**	1	-0.0596	0.7493	0.5662	-0.9195	1.0084	0.7722
	2	-0.0574	0.7484	0.5659	-0.9192	1.0080	0.7707
	3	-0.0498	0.7459	0.5642	-0.9162	1.0077	0.7707
	4	-0.0499	0.7460	0.5638	-0.9224	1.0093	0.7712
	5	-0.0558	0.7481	0.5658	-0.9226	1.0095	0.7721
	6	-0.0498	0.7459	0.5631	-0.9434	1.0147	0.7744
	7	-0.0603	0.7497	0.5669	-0.9595	1.0191	0.7792
	8	-0.0539	0.7473	0.5631	-0.9163	1.0076	0.7728
	9	-0.0593	0.7491	0.5655	-0.9451	1.0146	0.7817
	10	-0.0597	0.7495	0.5664	-0.9591	1.0188	0.7760
	11	-0.0569	0.7484	0.5656	-0.9166	1.0077	0.7700
	12	-0.0653	0.7514	0.5667	-0.9460	1.0154	0.7750
	13	-0.0508	0.7462	0.5626	-0.9555	1.0179	0.7779
	14	-0.0576	0.7486	0.5637	-0.9374	1.0130	0.7741
	15	-0.0619	0.7501	0.5650	-0.9413	1.0137	0.7775
	16	-0.0667	0.7519	0.5688	-0.9258	1.0102	0.7718
	17	-0.0542	0.7473	0.5641	-0.9181	1.0080	0.7717
	18	-0.0502	0.7460	0.5636	-0.9250	1.0098	0.7713
	19	-0.0536	0.7472	0.5652	-0.9016	1.0038	0.7704
	20	-0.0504	0.7461	0.5643	-0.9570	1.0183	0.7751
**Scaffold_groupkfold_murcko_y_randomized**	1	-0.0572	0.7480	0.5649	-0.9769	1.0168	0.7825
	2	-0.0428	0.7435	0.5629	-0.9695	1.0165	0.7808
	3	-0.0442	0.7441	0.5601	-0.9697	1.0161	0.7811
	4	-0.0439	0.7438	0.5606	-0.9804	1.0181	0.7815
	5	-0.0356	0.7404	0.5585	-0.9866	1.0180	0.7826
	6	-0.0381	0.7418	0.5597	-0.9991	1.0236	0.7853
	7	-0.0439	0.7449	0.5631	-1.0119	1.0284	0.7910
	8	-0.0446	0.7435	0.5613	-0.9749	1.0161	0.7836
	9	-0.0416	0.7417	0.5586	-1.0091	1.0229	0.7912
	10	-0.0488	0.7452	0.5634	-1.0164	1.0277	0.7863
	11	-0.0359	0.7396	0.5573	-0.9882	1.0162	0.7795
	12	-0.0369	0.7410	0.5591	-1.0066	1.0241	0.7865
	13	-0.0332	0.7403	0.5589	-1.0059	1.0262	0.7884
	14	-0.0414	0.7434	0.5600	-0.9923	1.0220	0.7853
	15	-0.0526	0.7457	0.5627	-1.0026	1.0219	0.7874
	16	-0.0449	0.7445	0.5632	-0.9730	1.0186	0.7818
	17	-0.0417	0.7443	0.5625	-0.9571	1.0168	0.7828
	18	-0.0459	0.7445	0.5634	-0.9828	1.0186	0.7820
	19	-0.0469	0.7452	0.5628	-0.9547	1.0123	0.7809

	20	-0.0342	0.7402	0.5591	-1.0209	1.0273	0.7862

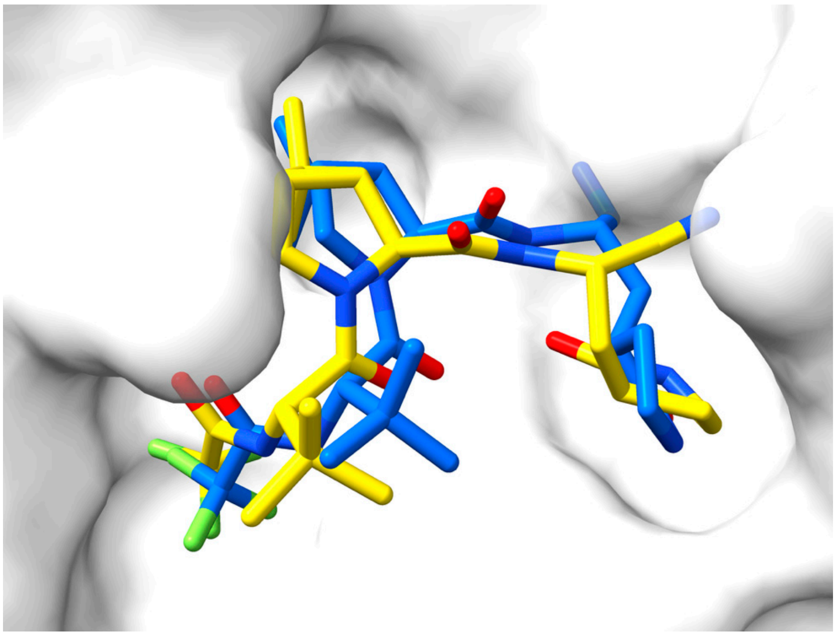

Figure S1. Docking validation and binding mode comparison of Nirmatrelvir analogues with MERS-CoV main protease. Superimposition of the crystallographic ligand (blue) and the re-docked pose (yellow) within the active site of the MERS-CoV main protease. The RMSD calculated over 35 corresponding atom pairs was 5.6 Å, indicating that the re-docked pose occupies the same binding region but shows conformational deviation from the crystallographic binding orientation.

Website to predict Binding score:

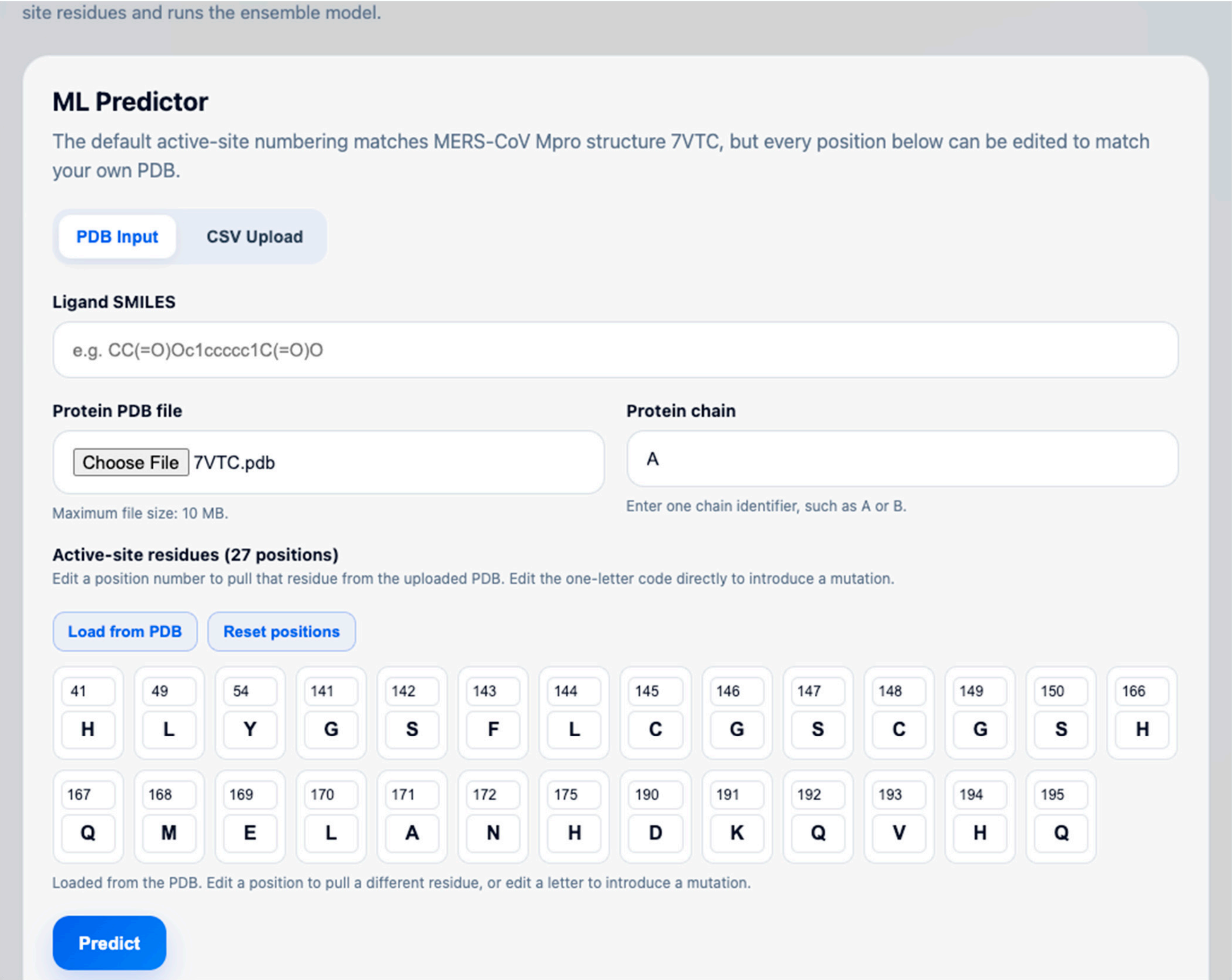

Figure S2: Web interface to predict binding score values (https://sites.google.com/view/shahidulislamlab/utilities/binding-affinity-predictor)

Codes related to the model used to predict Binding score:

All the codes relevant to this study are stored in GitHub under Binding-affinity-prediction repository in the following link: https://github.com/Dr-Islam-Lab-Group/Binding-affinity-prediction.
